# Combining chains of Bayesian models with Markov melding

**DOI:** 10.1214/22-BA1327

**Published:** 2023-09

**Authors:** Andrew A. Manderson, Robert J. B. Goudie

**Affiliations:** * MRC Biostatistics Unit, University of Cambridge, United Kingdom, and The Alan Turing Institute; † MRC Biostatistics Unit, University of Cambridge, United Kingdom

**Keywords:** Combining models, Markov melding, Bayesian graphical models, Multi-stage estimation, Model/data integration, Integrated population model

## Abstract

A challenge for practitioners of Bayesian inference is specifying a model that incorporates multiple relevant, heterogeneous data sets. It may be easier to instead specify distinct submodels for each source of data, then join the submodels together. We consider chains of submodels, where submodels directly relate to their neighbours via common quantities which may be parameters or deterministic functions thereof. We propose *chained Markov melding*, an extension of Markov melding, a generic method to combine chains of submodels into a joint model. One challenge we address is appropriately capturing the prior dependence between common quantities within a submodel, whilst also reconciling differences in priors for the same common quantity between two adjacent submodels. Estimating the posterior of the resulting overall joint model is also challenging, so we describe a sampler that uses the chain structure to incorporate information contained in the submodels in multiple stages, possibly in parallel. We demonstrate our methodology using two examples. The first example considers an ecological integrated population model, where multiple data sets are required to accurately estimate population immigration and reproduction rates. We also consider a joint longitudinal and time-to-event model with uncertain, submodel-derived event times. Chained Markov melding is a conceptually appealing approach to integrating submodels in these settings.

## 1 Introduction

The Bayesian philosophy is appealing in part because the posterior distribution quantifies all sources of uncertainty. However, a joint model for all data and parameters is a prerequisite to posterior inference, and in situations where multiple, heterogeneous sources of data are available, specifying such a joint model is a formidable task. Models that consider such data are necessary to describe complex phenomena at a useful precision. One possible approach begins by specifying individual submodels for each source of data. These submodels could guide the statistician when directly specifying the joint model, but to use the submodels only informally seems wasteful. Instead, it may be preferable to construct a joint model by formally joining the individual submodels together.

Some specific forms of combining data are well established. Meta-analyses and evidence synthesis methods are widely used to summarise data, often using hierarchical models (Ades and Sutton, 2006; Presanis et al., 2014). Outside of the statistical literature, a common name for combining multiple data is *data fusion* (Lahat et al., 2015; Kedem et al., 2017), though there are many distinct methods that fall under this general name. Interest in integrating data is not just methodological; applied researchers often collect multiple disparate data sets, or data of different modalities, and wish to combine them. For example, to estimate SARS-CoV-2 positivity Donnat et al. (2020) build an intricate hierarchical model that integrates both testing data and self-reported questionnaire data, and Parsons et al. (2021) specify a hierarchical model of similar complexity to estimate the number of injecting drug users in Ukraine. Both applications specify Bayesian models with data-specific components, which are united in a hierarchical manner. In conservation ecology, *integrated population models* (IPMs) (Besbeas et al., 2002; Brooks et al., 2004; Schaub and Abadi, 2011; Maunder and Punt, 2013; Zipkin and Saunders, 2018) are used to estimate population dynamics, e.g. reproduction and immigration rates, using multiple data on the same population. Such data have standard models associated with them, such as the Cormack-Jolly-Seber model (Lebreton et al., 1992) for capture-recapture data, and the IPM serves as the framework in which the standard models are combined. More generally, the applications we list illustrate the importance of generic, flexible methods for combining data to applied researchers.

*Markov melding* (Goudie et al., 2019) is a general statistical methodology for combining submodels. Specifically, it considers *M* submodels that share some common quantity *ϕ*, with each of the *m* = 1, …, *M* submodels possessing distinct parameters *ψ_m_*, data *Y_m_*, and form p_*m*_(*ϕ, ψ_m_, Y_m_*). Goudie et al. (2019) then propose to combine the submodels into a joint model, denoted p_meld_(*ϕ, ψ*_1_, …, *ψ_M_*, *Y*_1_, …, *Y_M_*). However, it is unclear how to integrate models where there is no single quantity *ϕ* common to all submodels, such as for submodels that are linked in a chain structure.

We propose an extension to Markov melding, which we call *chained Markov melding*^1^, which facilitates the combination of *M* submodels that are in a chain structure. For example, when *M* = 3 we address the case in which submodel 1 and 2 share a common quantity *ϕ*_1∩2_, and submodel 2 and 3 share a different quantity *ϕ*_2∩3_. Our extension addresses previously unconsidered complications including the distinct domains (and possibly supports) of the common quantities, and the desire to capture possible prior correlation between them. Two examples serve to illustrate our methodology, which we introduce in the following section. The computational effort required to fit a complex, multi-response model is a burden to the model development process. We propose a multi-stage posterior estimation method that exploits the properties of our chained melded model to reduce this burden. We can parallelise aspects of the computation across the submodels, using less computationally expensive techniques for some submodels. Reusing existing software implementations of submodels, and subposterior samples where available, is also possible. Multi-stage samplers can aid in understanding the contribution of each submodel to the final posterior, and are used in many applied settings, including hierarchical modelling (Lunn et al., 2013) and joint models (Mauff et al., 2020).

One contribution of our work is to clarify the informal process commonly used in applied analyses of summarising and/or approximating submodels for use in subsequent analyses. The two most common approximation strategies seem to be (i) approximating the subposterior of the common quantity with a normal distribution for use in subsequent models (see, e.g. Jackson and White, 2018; Nicholson et al., 2021) and (ii) taking only a point estimate of the subposterior, and treating it as a known value in further models. These strategies may, but not always, produce acceptable approximations to the chained melded model. Both the chained melded model and these approximation strategies are examples of ‘multi-phase’ and ‘multi-source’ inference (Meng, 2014), with the melding approach most comprehensively accounting for uncertainty.

### 1.1 Example introduction

In this section we provide a high-level overview of two applications that require integrating a chain of submodels, with more details in Sections 4 and 5. Our first example decomposes a joint model into its constituent submodels and rejoins them. This simple situation allows us to compare the output from the chained melding process to the complete joint model, and is meant to illustrate both the ‘chain-of-submodels’ notion and the mechanics of chained melding. The second example is a realistic and complex setting in which the combining of submodels without chained Markov melding is nonobvious. Our comparator is the common technique of summarising previously considered submodels with point estimates, and demonstrates the importance of fully accounting for uncertainty.

#### An integrated population model for little owls

**Figure 1:**
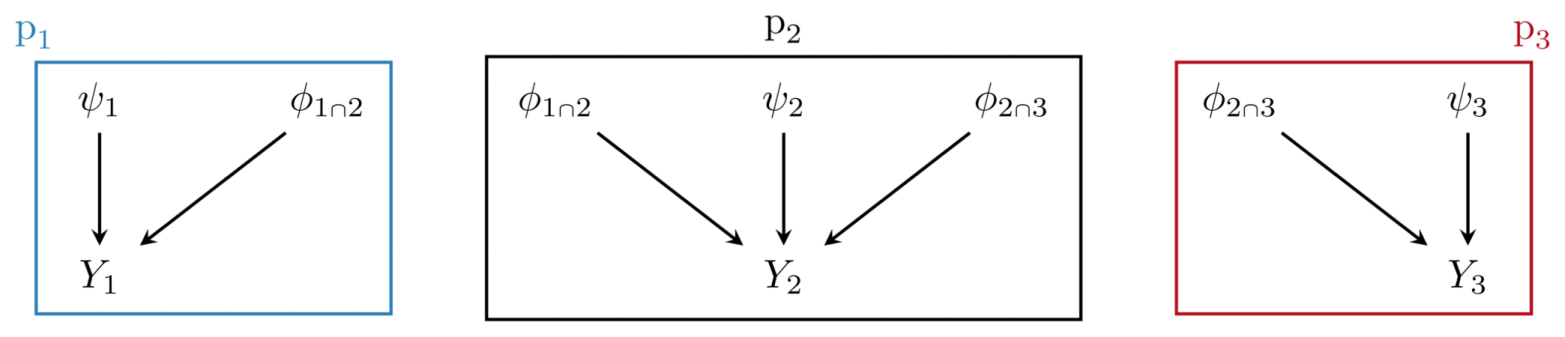
A simplified DAG of the integrated population model (IPM) for the little owls. The capture-recapture submodel (p_1_) is surrounded by the blue line, the population count submodel (p_2_) by the black line, and the fecundity submodel (p_3_) by the red line. The capture-recapture and population count submodels share parameters affecting the juvenile and adult survival rate (*ϕ*_1∩2_), whilst the parameter for fecundity is common to both the population count and fecundity submodels (*ϕ*_2∩3_). The combination of all the submodels forms the IPM.

Integrated population models (IPMs) (Zipkin and Saunders, 2018) combine multiple data to estimate key quantities governing the dynamics of a specific population. Schaub et al. (2006) and Abadi et al. (2010) used an IPM to estimate fecundity, immigration, and yearly survival rates for a population of little owls. These authors collect and model three types of data, illustrated in Figure 1. Capture-recapture data *Y*_1_, and associated capture-recapture submodel p_1_(*ϕ*_1∩2_, *ψ*_1_, *Y*_1_), are acquired by capturing and tagging owls each year, and then counting the number of tagged individuals recaptured in subsequent years. Population counts *Y*_2_ are obtained by observing the number of occupied nesting sites, and are modelled in p_2_(*ϕ*_1∩2_, *ϕ*_2∩3_, *ψ*_2_, *Y*_2_). Finally, nest-record data *Y*_3_ counts both the number of reproductive successes and possible breading pairs, and is associated with a submodel for fecundity p_3_(*ϕ*_2∩3_, *ψ*_3_, *Y*_3_). The population count model p_2_ shares the parameter *ϕ*_1∩2_ with the capture-recapture model p_1_, and the parameter *ϕ*_2∩3_ with the fecundity model p_3_; each of the *m* = 1, 2, 3 submodels has distinct, submodel-specific parameters *ψ_m_*. No single source of data is sufficient to estimate all quantities of interest, so it is necessary to integrate the three submodels into a single joint model to produce acceptably precise estimates of fecundity and immigration rates. We will show that the chained Markov melding framework developed in Section 2 encapsulates the process of integrating these submodels, producing results that are concordant with the original joint IPM.

#### Survival analysis with time varying covariates and uncertain event times

Our second example considers the time to onset of respiratory failure (RF) amongst patients in intensive care units, and factors that influence the onset of RF. A patient can be said to be experiencing RF if the ratio of the partial pressure of arterial blood oxygen (PaO_2_) to the faction of inspired oxygen (FiO_2_) is less than 300mmHg (The ARDS Definition Task Force, 2012), though this is not the only definition of RF. Patients’ PaO_2_/FiO_2_ (P/F) ratios are typically measured only a few times a day. The relative infrequency of P/F ratio data, when combined with the intrinsic variability in each individual’s blood oxygen level, results in significant uncertainty in about the time of onset of RF.

Factors that influence the time to onset of RF are both longitudinal and time invariant. Both types of data can be considered in *joint models* (Rizopoulos, 2012), which are composed of two distinct submodels, one for each data type. However, existing joint models are not able to incorporate the uncertainty surrounding the event time, which may result in overconfident and/or biased estimates of the parameters in the joint model.

**Figure 2:**
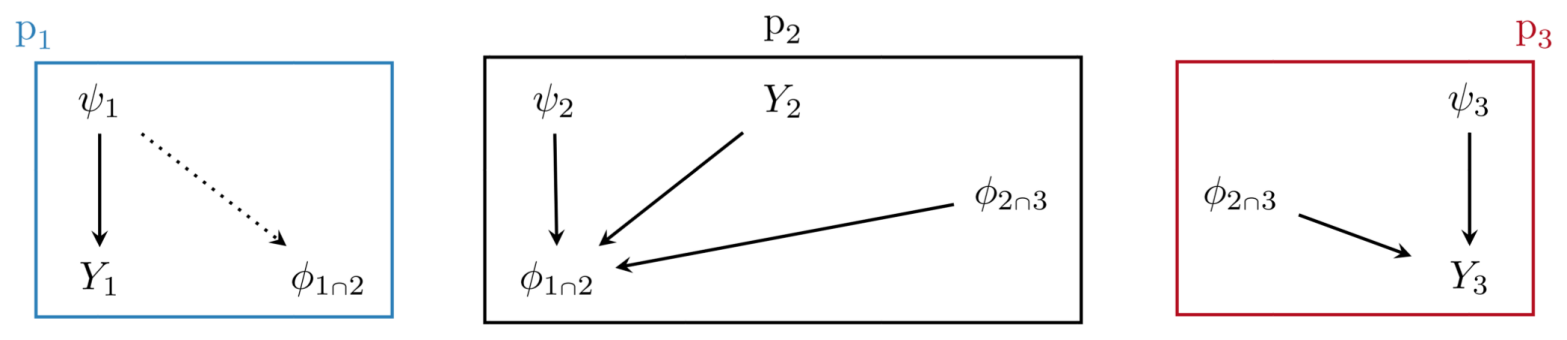
A simplified DAG of the submodels considered in the survival analysis example. The event time submodel p_1_ defines the event time *ϕ*_1∩2_ as noninvertible function of the other model parameters (denoted by the dotted line), whilst the survival submodel p_2_ considers *ϕ*_1∩2_ as the response. The longitudinal submodel p_3_ has parameters *ϕ*_2∩3_ in common with the survival submodel.

Chained Markov melding offers a conceptually straightforward, Bayesian approach to incorporating uncertain event times into joint models. Specifically, we consider the event time as a submodel-derived quantity from a hierarchical regression model akin to Lu and Meeker (1993). We call this submodel the *uncertain event time* submodel and denote it p_1_(*ϕ*_1∩2_, *ψ*_1_, *Y*_1_), where *ϕ*_1∩2_ incorporates the event time. The survival submodel p_2_(*ϕ*_1∩2_, *ϕ*_2∩3_, *ψ*_2_, *Y*_2_) uses the event time within *ϕ*_1∩2_, the common quantity, as the response. We treat the longitudinal submodel, p_3_(*ϕ*_2∩3_, *ψ*_3_, *Y*_3_), separately from the survival submodel, as is common in two-stage joint modelling (Mauff et al., 2020), and denote the subject-specific parameters that also appear in the survival model as *ϕ*_2∩3_. Each of the *m* = 1, 2, 3 has submodel-specific data *Y_m_* and parameters *ψ_m_*. The high level submodel relationships are displayed as a DAG in Figure 2.

It is in examples such as this one that we foresee the most use for chained Markov melding; a fully Bayesian approach is desired and the submodels are nontrivial in complexity, with no previously existing or obvious joint model.

### 1.2 Markov melding

We now review Markov melding (Goudie et al., 2019) before detailing our proposed extension. As noted in the introduction, Markov melding is a method for combining *M* submodels p_1_(*ϕ, ψ*_1_, *Y*_1_), …, p_*M*_ (*ϕ, ψ_M_, Y_M_*) which share the same *ϕ*. When the sub-model prior marginals p_*m*_(*ϕ*) are identical, i.e. p_*m*_(*ϕ*) = p(*ϕ*) for all *m*, it is possible to combine the submodels using *Markov combination* (Dawid and Lauritzen, 1993; Massa and Lauritzen, 2010)

(1) $$\begin{array}{ll} {\text{p}}_{\text{comb}}\left(\phi ,\psi_{1},\ldots ,\psi_{M},Y_{1},\ldots ,Y_{M}\right) & =\text{p}(\phi )\prod_{m=1}^{M}{\text{p}}_{m}\left(\psi_{m},Y_{m}|\phi \right),\\ & =\frac{\prod_{m=1}^{M}{\text{p}}_{m}\left(\phi ,\;\psi_{m},\;Y_{m}\right)}{\text{p}{\left(\phi \right)}^{M-1}}. \end{array}$$

Markov combination is not immediately applicable when submodel prior marginals are distinct, so Goudie *et al*. define a *marginal replacement* procedure, where individual submodel prior marginals are replaced with a common marginal p_pool_(*ϕ*) = *h*(p_1_(*ϕ*), …, p_*M*_(*ϕ*)) which is the result of a pooling function *h* that appropriately summarises all prior marginals (the choice of which is described below). The result of marginal replacement is

(2) $${\text{p}}_{\text{repl},m}\left(\phi ,\psi_{m},Y_{m}\right)={\text{p}}_{\text{pool}}(\phi )\frac{{\text{p}}_{m}\left(\phi ,\psi_{m},Y_{m}\right)}{{\text{p}}_{m}(\phi )}.$$

Goudie *et al*. show that p_repl*,m*_(*ϕ, ψ_m_, Y_m_*) minimises the Kullback–Leibler (KL) divergence between a distribution q(*ϕ, ψ_m_, Y_m_*) and p_*m*_(*ϕ, ψ_m_, Y_m_*) under the constraint that q(*ϕ*) = p_pool_(*ϕ*), and that marginal replacement is valid when *ϕ* is a deterministic function of the other parameters in submodel *m*. Markov melding joins the submodels via the Markov combination of the marginally replaced submodels

(3) $$\begin{array}{ll} {\text{p}}_{\text{meld}}\left(\phi ,\psi_{1},\ldots ,\psi_{M},Y_{1},\ldots ,Y_{M}\right) & ={\text{p}}_{\text{pool}}(\phi )\prod_{m=1}^{M}{\text{p}}_{\text{repl},m}\left(\psi_{m},Y_{m}|\phi \right),\\ & ={\text{p}}_{\text{pool}}(\phi )\prod_{m=1}^{M}\frac{{\text{p}}_{m}\left(\phi ,\psi_{m},Y_{m}\right)}{{\text{p}}_{m}(\phi )}. \end{array}$$

#### Pooled prior

Goudie *et al*. proposed forming p_pool_(*ϕ*) using linear or logarithmic prior pooling (O’Hagan et al., 2006; Genest et al., 1986)

(4) $${\text{p}}_{\text{pool},\;\text{lin}}(\phi )=\frac{1}{K_{\text{lin}}(\lambda )}\sum_{m=1}^{M}\lambda_{m}{\text{p}}_{m}(\phi ),\;K_{\text{lin}}(\lambda )=\int^{\text{​}}\sum_{m=1}^{M}\lambda_{m}{\text{p}}_{m}(\phi )\text{d}\phi ,$$

(5) $${\text{p}}_{\text{pool},\;\text{log}}(\phi )=\frac{1}{K_{\text{log}}(\lambda )}\prod_{m=1}^{M}{\text{p}}_{m}{\left(\phi \right)}^{\lambda_{m}},\;K_{\text{log}}(\lambda )=\int^{\text{​}}\prod_{m=1}^{M}{\text{p}}_{m}{\left(\phi \right)}^{\lambda_{m}}\text{d}\phi ,$$

where λ = (λ_1_, …, λ_*M*_) are nonnegative weights, which are chosen subjectively to ensure p_pool_(*ϕ*) appropriately represents prior knowledge about the common quantity. Two special cases of pooling are of particular interest. *Product of experts (PoE) pooling* (Hinton, 2002) is a special case of logarithmic pooling that occurs when λ_*m*_ = 1 for all *m*. *Dictatorial pooling* is a special case of either pooling method when λ_*m*_′ = 1 and, for all *m* ≠ *m^′^*, λ_*m*_ = 0.

## 2 Chained model specification

Consider *m* = 1, …, *M* submodels each with data *Y_m_* and parameters *θ_m_* denoted p_*m*_(*θ_m_, Y_m_*), with *M ≥* 3. We assume that the submodels are connected in a manner akin to a chain and so can be ordered such that only ‘adjacent’ submodels in the chain have parameters in common. Specifically we assume that submodels *m* and *m* + 1 have some parameter *ϕ*_*m*∩*m*+1_ in common for *m* = 1, …, *M* − 1. For notational convenience define *ϕ*_1_ = *ϕ*_1∩2_, *ϕ_M_* = *ϕ_M−_*_1*∩M*_ and *ϕ_m_* = (*ϕ*_*m*−__1∩*m*_, ϕ_*m*∩*m*+1_) for *m* = 2, …, *M* −1, so that *ϕ_m_ ⊆ θ_m_* denotes the parameters in model *m* shared with another submodel. The submodel-specific parameters of submodel *m* are thus *ψ_m_* = *θ_m_ \ ϕ_m_*. Define the vector of all common quantities $\phi =\cup_{m=1}^{M}\phi_{m}=\left(\phi_{1\cap^{\text{​}}2},\phi_{2\cap^{\text{​}}3},\ldots ,\phi_{M-1\cap^{\text{​}}M}\right)$ so that all elements in *ϕ* are unique. Further denote by *ϕ_−m_* the subvector of *ϕ* excluding the *m*^th^ element. It will also be convenient to define *ψ* = (*ψ*_1_, …, *ψ_M_*) and likewise ***Y*** = (*Y*_1_, …, *Y_M_*). Note that all components of *ϕ, ψ* and ***Y*** may themselves be multivariate. Additionally, because *ϕ_m∩m_*_+1_ may be a deterministic function of either *θ_m_* or *θ_m_*_+1_ we refer to *ϕ*_*m*∩*m*__+1_ as a common parameter or a common quantity as appropriate.

All submodels, and marginal and conditional distributions thereof, have density functions that are assumed to exist and integrate to one. When considering conditional distributions we assume that the parameter being conditioned on has support in the relevant region. We define the *m*^th^ *subposterior* as p_*m*_(*ϕ_m_, ψ_m_ | Y_m_*).

### 2.1 Extending marginal replacement

We now define the chained melded model by extending the marginal replacement procedure to submodels linked in a chain-like way. The proposed chained marginal replacement operation modifies the submodels to enforce a common prior for *ϕ*. This consistency allows us to employ Markov combination to unite the submodels.

Specifically, the *m*^th^ marginally replaced submodel is

(6) $${\text{p}}_{\text{repl},m}\left(\phi ,\psi_{m},Y_{m}\right)={\text{p}}_{\text{pool}}(\phi ){\text{p}}_{m}\left(\psi_{m},Y_{m}|\phi \right)={\text{p}}_{\text{pool}}(\phi )\frac{{\text{p}}_{m}\left(\phi_{m},\psi_{m},Y_{m}\right)}{{\text{p}}_{m}\left(\phi_{m}\right)},$$

where p_pool_(*ϕ*) = *g*(p_1_(*ϕ*_1_), p_2_(*ϕ*_2_), …, p_*m*_(*ϕ_m_*)) is a pooling function that appropriately summarises all submodel prior marginals. The second equality in Equation (6) is because of the conditional independence (*ψ_m_*, *Y_m_* ⫫ *ϕ*_−*m*_) | *ϕ_m_* that exists due to the chained relationship between submodels. It is important to note that p_repl,*m*_(*ϕ, ψ_m_, Y_m_*) is defined on a larger parameter space than p_*m*_(*ϕ_m_, ψ_m_, Y_m_*), as it includes *ϕ_−m_*.

Define p_repl,*m*_(*ϕ_m_*, *ψ_m_*, *Y_m_*) = ∫ p_repl,*m*_(*ϕ, ψ_m_, Y_m_*)d*ϕ_−m_*. Each marginally replaced submodel, as defined in Equation (6), minimises the following KL divergence^2^

(7) $${\text{p}}_{\text{repl},m}\left(\phi_{m},\psi_{m},Y_{m}\right)=\underset{\text{q}}{\text{arg}\;\text{min}}\left\{D_{\text{KL}}\left(\text{q}\;||\;{\text{p}}_{m}\right)|\text{q}\left(\phi_{m}\right)={\text{p}}_{\text{pool}}\left(\phi_{m}\right)\;\text{for}\;\text{all}\;\phi_{m}\right\},$$

where p_pool_(*ϕ*_m_) = ∫ p_pool_(*ϕ*)d*ϕ*_−*m*_. We can thus interpret p_repl,*m*_(*ϕ_m_, ψ_m_, Y_m_*) as a minimally modified p_*m*_(*ϕ_m_*, *ψ_m_*, *Y_m_*) which admits p_pool_(*ϕ_m_*) as a marginal. Note that it is the combination of p_repl,*m*_(*ϕ_m_, ψ_m_, Y_m_*) and p_pool_(*ϕ_−m_ | ϕ_m_*) that uniquely determine (6).

We form the chained melded model by taking the Markov combination of the marginally replaced submodels

(8) $${\text{p}}_{\text{meld}}(\phi ,\psi ,Y)={\text{p}}_{\text{pool}}(\phi )\prod_{m=1}^{M}{\text{p}}_{\text{repl},m}\left(\psi_{m},Y_{m}|\phi \right),$$

(9) $$={\text{p}}_{\text{pool}}(\phi )\prod_{m=1}^{M}\frac{{\text{p}}_{m}\left(\phi_{m},\psi_{m},Y_{m}\right)}{{\text{p}}_{m}\left(\phi_{m}\right)}.$$

Rewriting (9) in terms of *ϕ*_*m*∩*m*+1_ for *m* = 1, …, *M* − 1 yields

(10) $${\text{p}}_{\text{meld}}(\phi ,\psi ,Y)={\text{p}}_{\text{pool}}(\phi )\frac{{\text{p}}_{1}\left(\phi_{1\cap^{\text{​}}2},\psi_{1},Y_{1}\right)}{{\text{p}}_{1}\left(\phi_{1\cap^{\text{​}}2}\right)}\frac{{\text{p}}_{M}\left(\phi_{M-1\cap^{\text{​}}M},\psi_{M},Y_{M}\right)}{{\text{p}}_{M}\left(\phi_{M-1\cap^{\text{​}}M}\right)},\times \prod_{m=2}^{M-1}\left(\frac{{\text{p}}_{m}\left(\phi_{m-1\cap^{\text{​}}m},\phi_{m\cap^{\text{​}}m+1},\psi_{m},Y_{m}\right)}{{\text{p}}_{m}\left(\phi_{m-1\cap^{\text{​}}m},\phi_{m\cap^{\text{​}}m+1}\right)}\right).$$

Finally, we use *chained melded posterior* p_meld_(*ϕ, ψ | **Y***) ∝ p_meld_(*ϕ, ψ, **Y***) to refer to posterior of the chained melded model conditioned on all data.

### 2.2 Pooled prior

Specifying (9) requires a joint prior for *ϕ*. As in Markov melding we form the joint prior by pooling the marginal priors, selecting a pooling function *g* that appropriately represents prior knowledge about the common quantities. We define p_pool_(*ϕ*) as a generic function of all prior marginals

(11) $${\text{p}}_{\text{pool}}(\phi )=g\left({\text{p}}_{1}\left(\phi_{1}\right),\;{\text{p}}_{2}\left(\phi_{2}\right),\ldots ,{\text{p}}_{M}\left(\phi_{M}\right)\right),$$

(12) $$=g\left({\text{p}}_{1}\left(\phi_{1\cap 2}\right),\;{\text{p}}_{2}\left(\phi_{1\cap 2},\phi_{2\cap 3}\right),\ldots ,{\text{p}}_{M}\left(\phi_{M-1\cap M}\right)\right),$$

because we do not always wish to assume independence between the components of *ϕ*.

Two special cases of Equation (12) are noteworthy. Firstly, if all components of *ϕ* are independent, then we can form p_pool_(*ϕ*) as the product of *M* − 1 standard pooling functions *h_m_* defined in Section 1.2

(13) $${\text{p}}_{\text{pool}}(\phi )=\prod_{m=1}^{M-1}{\text{p}}_{\text{pool},m}\left(\phi_{m\cap m+1}\right),$$

(14) $${\text{p}}_{\text{pool},m}\left(\phi_{m\cap m+1}\right)=h_{m}\left({\text{p}}_{m}\left(\phi_{m\cap m+1}\right),{\text{p}}_{m+1}\left(\phi_{m\cap m+1}\right)\right).$$

A second case, in between complete dependence (12) and independence (14), is that if p_*m*_(*ϕ_m−_*_1*∩m*_, *ϕ*_*m∩m*+1_) = p_*m*_(*ϕ_m−_*_1*∩m*_)p_*m*_(*ϕ_m∩m_*_+1_) then we can define

(15) $${\text{p}}_{\text{pool}}(\phi )=g_{1}\left({\text{p}}_{1}\left(\phi_{1\cap 2}\right),\ldots ,{\text{p}}_{m}\left(\phi_{m-1\cap m}\right)\right)g_{2}\left({\text{p}}_{m}\left(\phi_{m\cap m+1}\right),\ldots ,{\text{p}}_{M}\left(\phi_{M}\right)\right),$$

without any additional assumptions. That is, if any two consecutive components of *ϕ* are independent in the submodel containing both of them, we can divide the pooled prior specification problem into two pooling functions. The smaller number of arguments to *g*_1_ and *g*_2_ make it easier to choose appropriate forms for those functions.

Selecting a specific form of *g* is not trivial given the many choices of functional form and pooling weights (the latter of which we discuss momentarily). One complication is that standard linear and logarithmic pooling, as defined in Equations (4) and (5), are not immediately applicable when the submodel marginal distributions consider different quantities. We now propose extensions to logarithmic, linear, and dictatorial pooling for use in the case of chained melding.

#### Chained logarithmic pooling

Extending logarithmic pooling for chained Markov melding is straightforward. We define the logarithmically pooled prior to be

(16) $${\text{p}}_{\text{pool},\;\text{log}}(\phi )=\frac{1}{K_{\text{log}}(\lambda )}\prod_{m=1}^{M}{\text{p}}_{m}{\left(\phi_{m}\right)}^{\lambda_{m}},$$

with $K_{\text{log}}(\lambda )=\int \prod_{m=1}^{M}{\text{p}}_{m}{\left(\phi_{m}\right)}^{\lambda_{m}}\text{d}\phi$ for nonnegative weight vector *λ* = (*λ*_1_, …, *λ_M_*) and $\sum_{m=1}^{M}\lambda_{m}\geq 1$. Note that (16) does not imply independence between the elements of *ϕ* because

(17) $$\prod_{m=1}^{M}{\text{p}}_{m}{\left(\phi_{m}\right)}^{\lambda_{m}}={\text{p}}_{1}{\left(\phi_{1\cap 2}\right)}^{\lambda_{1}}\prod_{m=2}^{M-1}\left({\text{p}}_{m}{\left(\phi_{m-1\cap m},\phi_{m\cap m+1}\right)}^{\lambda_{m}}\right)\;{\text{p}}_{M}{\left(\phi_{M-1\cap M}\right)}^{\lambda_{M}}.$$

When *λ*_1_ = *λ*_2_ = … = *λ_M_* = 1 we obtain a special case which we call product-of-experts (PoE) pooling (Hinton, 2002).

#### Chained linear pooling

Our generalisation of linear pooling to handle marginals of different quantities is a two step procedure. The first step forms intermediary pooling densities via standard linear pooling, using appropriate marginals of the relevant quantity

(18) $${\text{p}}_{\text{pool},m}\left(\phi_{m\cap m+1}\right)\propto \lambda_{m,1}{\text{p}}_{m}\left(\phi_{m\cap m+1}\right)+\lambda_{m,2}{\text{p}}_{m+1}(\phi_{m\cap m+1}),$$

where *λ_m_* = (*λ*_*m*,1_, *λ*_*m*,2_) are nonnegative pooling weights, and for *m* = 2, …, *M* − 1

(19) $${\text{p}}_{m}\left(\phi_{m\cap m+1}\right)=\int {\text{p}}_{m}\left(\phi_{m-1\cap m},\phi_{m\cap m+1}\right)\text{d}\phi_{m-1\cap m}.$$

For *m* = 1 and *m* = *M* the relevant marginals are p_1_(*ϕ*_1∩2_) and p_*M*_(*ϕ_M−_*_1*∩M*_). In step two we form the pooled prior as the product of the intermediaries

(20) $${\text{p}}_{\text{pool},\;\text{lin}}(\phi )=\frac{1}{K_{\text{lin}}(\lambda )}\prod_{m=1}^{M-1}{\text{p}}_{\text{pool},m}\left(\phi_{m\cap m+1}\right),$$

with $K_{\text{lin}}(\lambda )=\int \prod_{m=1}^{M-1}{\text{p}}_{\text{pool},m}\left(\phi_{m\cap m+1}\right)\text{d}\phi$, for *λ* = (*λ*_1_, …, *λ_M_*). Clearly, this assumes prior independence amongst all components of *ϕ* which may be undesirable, particularly if this independence was not present under one or more of the submodel priors. We discuss extensions to linear pooling that enable prior dependence between the components of *ϕ* in Section 6.

#### Dictatorial pooling

Chained Markov melding does not admit a direct analogue to dictatorial pooling as defined in Section 1.2 because not all submodel prior marginals contain all common quantities. For example, consider the logarithmically pooled prior of Equation (16) with, say, the *m*^th^ entry in *λ* set to 1 and all others set to 0. This choice of *λ* results in p_pool_(*ϕ*) = p(*ϕ_m_*), which is flat for *ϕ_−m_*. It seems reasonable to require any generalisation of dictatorial pooling to result in a reasonable prior for all components in *ϕ*. Such a generalisation should also retain the original intention of dictatorial pooling, i.e. ‘*the authoritative prior for ϕ_m_ is* p_*m*_(*ϕ_m_*)’.

We propose two possible forms of dictatorial pooling that satisfy the aforementioned criteria. *Partial dictatorial pooling* enforces a single submodel prior for the relevant components of *ϕ*, with no restrictions on the pooling of the remaining components; and *complete dictatorial pooling* which requires selecting one of the two possible submodel priors for each component of *ϕ*.

Partial dictatorial pooling considers p_*m*_(*ϕ_m_*) as the authoritative prior for *ϕ_m_* = (*ϕ_m−_*_1∩*m*_, *ϕ*_*m*∩*m*+1_). This results in,

(21) $$\begin{array}{ll} {\text{p}}_{\text{pool},\text{dict}}(\phi )= & g_{1}\left({\text{p}}_{1}\left(\phi_{1\cap 2}\right),\ldots ,{\text{p}}_{m-1}\left(\phi_{m-2\cap m-1}\right)\right)\\ & \begin{array}{l} \times {\text{p}}_{m}\left(\phi_{m-1\cap m},\phi_{m\cap m+1}\right)\\ \times g_{2}\left({\text{p}}_{m+1}\left(\phi_{m+1\cap m+2}\right),\ldots ,{\text{p}}_{M}\left(\phi_{M-1\cap M}\right)\right), \end{array} \end{array}$$

where *g*_1_ and *g*_2_ are linear or logarithmic pooling functions as desired^3^.

Complete dictatorial pooling requires the marginal pooled prior for each component in *ϕ* to be chosen solely on the basis of only one of the two priors specified for it under the submodels. For *m* = 1, …, *M* − 1, the *m*^th^ marginal of the pooled prior is either

(22) $${\text{p}}_{\text{pool},\text{dict}}\left(\phi_{m\cap m+1}\right)≔\{\begin{array}{l} {\text{p}}_{m}\left(\phi_{m\cap m+1}\right)\;\text{or}\\ {\text{p}}_{m+1}(\phi_{m\cap m+1}). \end{array}$$

If two consecutive marginals are chosen to have the same submodel prior, then we wish to retain the dependence between *ϕ_m−_*_1∩*m*_ and *ϕ_m∩m_*_+1_ present in p_*m*_. We thus redefine consecutive terms so that

(23) $$\begin{array}{l} {\text{p}}_{\text{pool},\text{dict}}\left(\phi_{m-1\cap m}\right){\text{p}}_{\text{pool},\text{dict}}\left(\phi_{m\cap m+1}\right)=p_{m}\left(\phi_{m-1\cap m}\right)p_{m}\left(\phi_{m\cap m+1}\right)\;\;\;\;\;\;\;\text{(FROM}\;\text{EQ}.\text{(22);}\\ {\text{p}}_{\text{pool},\text{dict}}\left(\phi_{m-1\cap m}\right){\text{p}}_{\text{pool},\text{dict}}\left(\phi_{m\cap m+1}\right)≔{\text{p}}_{m}(\phi_{m-1\cap m},\phi_{m\cap m+1}).\;\;\;\;\;\;\;\;\;\;\;\;\;\;\;\;\;\;\text{(REDEFINED)} \end{array}$$

The complete dictatorially pooled prior is thus

(24) $${\text{p}}_{\text{pool},\text{dict}}(\phi )=\prod_{m=1}^{M-1}{\text{p}}_{\text{pool},\text{dict}}(\phi_{m\cap m+1}),$$

where, subject to the potential modification in Equation (23), the terms in the product are as defined in Equation (22). For example, if *M* = 5 and we wish to ignore p_2_ and p_4_ when constructing the pooled prior and instead associate *ϕ*_1∩2_ with p_1_, both *ϕ*_2∩3_ and *ϕ*_3∩4_ with p_3_, and *ϕ*_4∩5_ with p_5_, then

(25) $$\begin{array}{ll} {\text{p}}_{\text{pool},\text{dict}}(\phi ) & =\overset{\text{APPLY}\;\text{EQ}.\;\text{(23)}}{\overset{︷}{{\text{p}}_{1,\text{dict}}\left(\phi_{1\cap 2}\right){\text{P}}_{3,\text{dict}}\left(\phi_{2\cap 3}\right){\text{p}}_{3,\text{dict}}\left(\phi_{3\cap 4}\right){\text{p}}_{5,\text{dict}}\left(\phi_{4\cap 5}\right)}}\\ & ={\text{p}}_{1}\left(\phi_{1\cap 2}\right){\text{p}}_{3}\left(\phi_{2\cap 3},\phi_{3\cap 4}\right){\text{p}}_{5}(\phi_{4\cap 5}). \end{array}$$

#### Pooling weights

**Figure 3:**
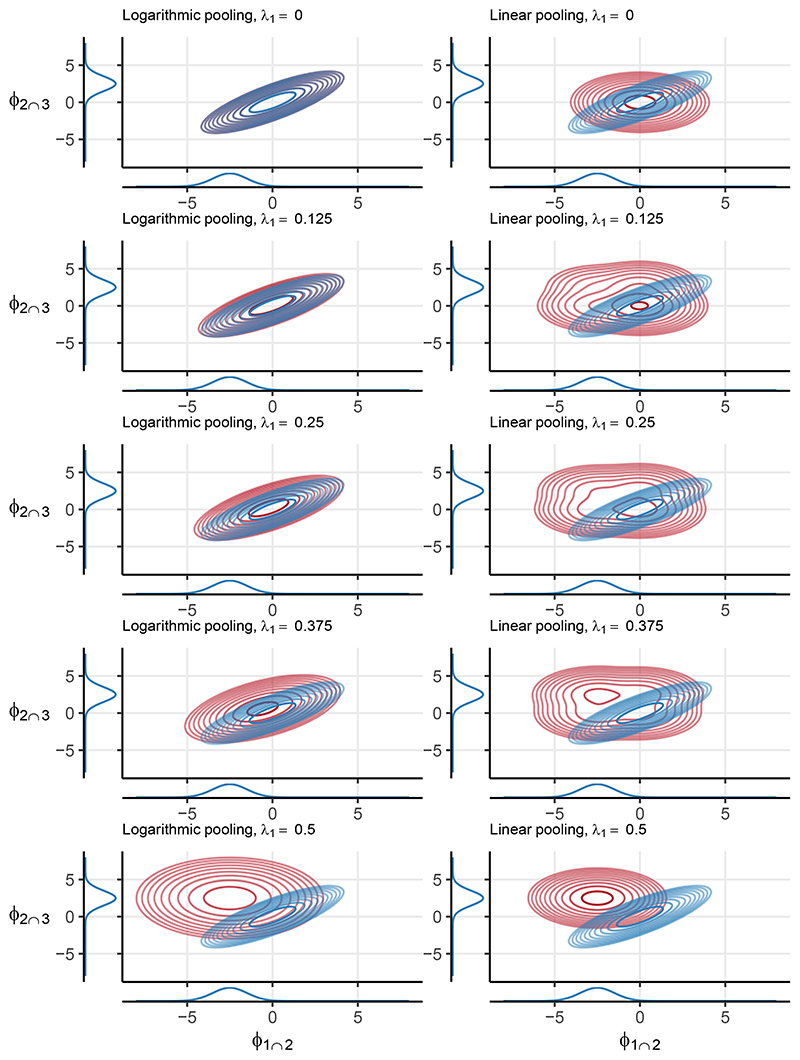
Contour plots of p_pool_(*ϕ*) (red) under logarithmic and linear pooling (left and right column respectively). The three original densities p_1_(*ϕ*_1∩2_), p_3_(*ϕ*_2∩3_) and p_2_(*ϕ*_1∩2_, *ϕ*_2∩3_) are shown in blue, with the univariate densities shown on the appropriate axis. The pooling weight parameter *λ*_1_ is indicated in the plot titles.

Choosing values for the pooling weights is an important step in specifying the pooled prior (Carvalho et al., 2022; Abbas, 2009; Rufo et al., 2012a,b). Because appropriate values for the weights depend on the submodels being pooled and the information available *a priori*, universal recommendations are impossible, so we illustrate the impact of different choices in a straightforward example. It is important that prior predictive visualisations of the pooled prior are produced (Gabry et al., 2019; Gelman et al., 2020) to guide the choice of pooling weights and ensure that the result suitably represents the available information. Figure 3 illustrates how *λ* and the choice of pooling method impacts p_pool_(*ϕ*) when pooling normal distributions. Specifically, we consider *M* = 3 submodels and pool

(26) $$\begin{array}{c} {\text{p}}_{1}\left(\phi_{1\cap 2}\right)=\text{N}\left(\phi_{1\cap 2};\mu_{1},\sigma_{1}^{2}\right),\;{\text{p}}_{3}\left(\phi_{2\cap 3}\right)=\text{N}\left(\phi_{2\cap 3};\mu_{3},\sigma_{3}^{2}\right),\\ {\text{p}}_{2}\left(\phi_{1\cap 2},\phi_{2\cap 3}\right)=\text{N}(\left[\begin{array}{l} \phi_{1\cap 2}\\ \phi_{2\cap 3} \end{array}\right];\left[\begin{array}{l} \mu_{2,1}\\ \mu_{2,2} \end{array}\right],\left[\begin{array}{cc} \sigma_{2}^{2} & \rho \sigma_{2}^{2}\\ \rho \sigma_{2}^{2} & \sigma_{2}^{2} \end{array}\right]), \end{array}$$

where N(*ϕ*; *μ, σ*^2^) is the normal density function with mean *μ* and variance *σ*^2^ (or covariance matrix where appropriate). The two dimensional density function p_2_ has an additional parameter *ρ*, which controls the intra-submodel marginal correlation. We set *μ*_1_ = −2.5, *μ*_2_ = [*μ*_2,1_ *μ*_2,2_]′ = [0 0]′, $\mu_{3}=2.5,\sigma_{1}^{2}=\sigma_{2}^{2}=\sigma_{3}^{2}=1$ and *ρ* = 0.8. In the logarithmic case we set *λ*_1_ = *λ*_3_ and parameterise *λ*_2_ = 1−2*λ*_1_, so that *λ*_1_ +*λ*_2_ +*λ*_3_ = 1 whilst limiting ourselves to varying only *λ*_1_. Similarly, in the linear case we set *λ*_1,1_ = *λ*_2,2_ = *λ*_1_ and *λ*_1,2_ = *λ*_2,1_ = 1 *−* 2*λ*_1_. We consider 5 evenly spaced values of *λ*_1_ *∈* [0, 0.5].

For both pooling methods, as the weight *λ*_1_ associated with models p_1_ and p_3_ increases, the relative contributions of p_1_(*ϕ*_1∩2_) and p_3_(*ϕ*_2∩3_) increase. Note the lack of correlation in p_pool_ under linear pooling (right column of Figure 3) due to Equation (20). A large, near-flat plateau is visible in the *λ*_1_ = 0.25 and *λ*_1_ = 0.375 cases, which is a result of the mixture of four, 2-D normal distributions that linear pooling produces in this example. The logarithmic pooling process produces a more concentrated prior for small values of *λ*_1_, and does not result in *a priori* independence between *ϕ*_1∩2_ and *ϕ*_2∩3_. Appendix A shows analytically that *λ*_2_ controls the quantity of correlation present in p_pool_ in this setting.

## 3 Posterior estimation

We now present a multi-stage MCMC method for generating samples from the melded posterior. Whilst the melded posterior is a standard Bayesian posterior and so can, in principle, be targeted using any suitable Monte Carlo method, in practice this may be cumbersome or infeasible. More specifically, it may be feasible to fit each submodel separately using standard methods, but when the submodels are combined – either through Markov melding, or by expanding the definition of one submodel to include another – the computation required to estimate the posterior in a single step poses an insurmountable barrier. In such settings we can employ multi-stage posterior estimation methods including Tom et al. (2010), Lunn et al. (2013), Hooten et al. (2019), and Mauff et al. (2020). We propose a multi-stage strategy that uses the chain-like relationship to both avoid evaluating all submodels simultaneously, and parallelise the computation required in the first stage to produce posterior samples in less time than an equivalent sequential method^4^. Avoiding concurrently evaluating all submodels also enables the reuse of existing software, minimising the need for custom submodel and/or sampler implementations.

We also describe an approximate method, where stage one submodels are summarised by normal distributions for use in stage two.

We consider the *M* = 3 case, as this setting includes both of our examples. Our approach can be extended to *M >* 3 settings, although we anticipate that it is unlikely to be suitable for large *M* settings. We discuss some of difficulties associated with generic, parallel methodology for efficient posterior sampling in Section 6.

### 3.1 Parallel sampler

**Figure 4:**
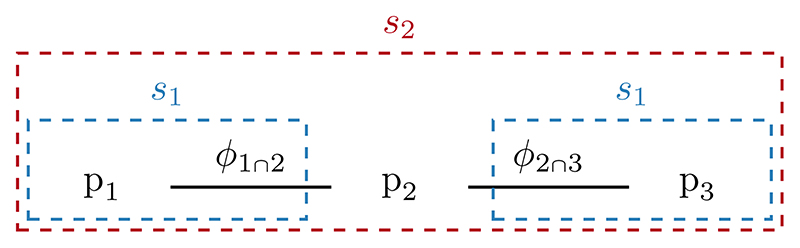
A graphical depiction of the submodels and their shared quantities, with the parallel sampling strategy overlaid. The stage one (*s*_1_) targets are surrounded by blue dashed lines, with the stage two (*s*_2_) target in red.

Our proposed strategy involves obtaining in stage one samples from submodels 1 and 3 in parallel. Stage two reuses these samples in a Metropolis-within-Gibbs sampler, which targets the full melded posterior. The stage specific targets are displayed in Figure 4.

The parallel sampler assumes that the pooled prior decomposes such that

(27) $${\text{p}}_{\text{pool}}(\phi )={\text{p}}_{\text{pool},1}\left(\phi_{1\cap 2}\right){\text{p}}_{\text{pool},2}\left(\phi_{1\cap 2},\phi_{2\cap 3}\right){\text{p}}_{\text{pool},3}\left(\phi_{2\cap 3}\right).$$

All pooled priors trivially satisfy (27) by assuming p_pool,1_(*ϕ*_1∩2_) and p_pool,3_(*ϕ*_2∩3_) are improper and/or flat distributions. Alternatively we may choose p_pool,1_(*ϕ*_1∩2_) = p_1_(*ϕ*_1∩2_) and p_pool,3_(*ϕ*_2∩3_) = p_3_(*ϕ*_2∩3_), with appropriate adjustments to p_pool,2_(*ϕ*_1∩2_, *ϕ*_2∩3_). This choice targets, in stage one, the subposteriors of p_1_ and p_3_ under their original prior for *ϕ*_1∩2_ and *ϕ*_2∩3_ respectively.

#### Stage one

Two independent, parallel sampling processes occur in stage one. Terms from the melded model that pertain to p_1_ and p_3_ are isolated

(28) $${\text{p}}_{\text{meld},1}\left(\phi_{1\cap 2},\psi_{1}|Y_{1}\right)\propto {\text{p}}_{\text{pool},1}\left(\phi_{1\cap 2}\right)\frac{{\text{p}}_{1}\left(\phi_{1\cap 2},\psi_{1},Y_{1}\right)}{{\text{p}}_{1}\left(\phi_{1\cap 2}\right)},$$

(29) $${\text{p}}_{\text{meld},3}\left(\phi_{2\cap^{\text{​}}3},\psi_{3}|Y_{3}\right)\propto {\text{p}}_{\text{pool},3}\left(\phi_{2\cap^{\text{​}}3}\right)\frac{{\text{p}}_{3}\left(\phi_{2\cap^{\text{​}}3},\psi_{3},Y_{3}\right)}{{\text{p}}_{3}\left(\phi_{2\cap^{\text{​}}3}\right)},$$

and targeted using standard MCMC methodology. Assuming that the stage one chains converge and after discarding warmup iterations –possibly thinning them, if within-chain correlation is high– we obtain *N*_1_ samples from ${\left\{{\left(\phi_{1\cap^{\text{​}}2},\psi_{1}\right)}_{n}\right\}}_{n=1}^{N_{1}}$ from p_meld,1_(*ϕ*_1∩2_, *ψ*_2_ *|* *Y*_1_), and *N*_3_ samples ${\left\{{\left(\phi_{2\cap^{\text{​}}3},\psi_{3}\right)}_{n}\right\}}_{n=1}^{N_{3}}$ from p_meld,3_(*ϕ*_2∩3_, *ψ*_3_ *| Y*_3_). For well mixing stage one Markov chains targeting the correct stationary distribution, and large values of *N*_1_ or *N*_3_, the stage one samples accurately approximate the subposteriors.

#### Stage two

Stage two targets the melded posterior of Equation (9) using a Metropolis-within-Gibbs sampler, where the proposal distributions are

(30) $$\phi_{1\cap^{\text{​}}2}^{*},\psi_{1}^{*}|\phi_{2\cap^{\text{​}}3},\psi_{2},\psi_{3}∼{\text{p}}_{\text{meld},1}\left(\phi_{1\cap^{\text{​}}2}^{*},\psi_{1}^{*}|Y_{1}\right),$$

(31) $$\phi_{2\cap^{\text{​}}3}^{*},\psi_{3}^{*}|\phi_{1\cap^{\text{​}}2},\psi_{1},\psi_{2}∼{\text{p}}_{\text{meld},3}\left(\phi_{2\cap^{\text{​}}3}^{*},\psi_{3}^{*}|Y_{3}\right),$$

(32) $$\psi_{2}^{*}\;|\;\phi_{1\cap 2},\phi_{2\cap 3,}\psi_{1},\psi_{3}∼\text{q}\left(\psi_{2}^{*}\;|\;\psi_{2}\right),$$

where $\text{q}\left(\psi_{2}^{*}|\psi_{2}\right)$ is a generic proposal distribution for *ψ*_2_. We draw an index $n_{1}^{*}$ uniformly from {1, …, *N*_1_} and use the corresponding value ${\left(\phi_{1\cap^{\text{​}}2}^{*},\psi_{1}^{*}\right)}_{n_{1}^{*}}$ as the proposal, doing likewise for $n_{3}^{*}$ and ${\left(\phi_{2\cap^{\text{​}}3}^{*},\psi_{3}^{*}\right)}_{n_{3}^{*}}$. The acceptance probabilities for these updates are

(33) $$\alpha \left({\left(\phi_{1\cap^{\text{​}}2}^{*},\psi_{1}^{*}\right)}_{n_{1}^{*}},{\left(\phi_{1\cap^{\text{​}}2},\psi_{1}\right)}_{n_{1}}\right)=\frac{{\text{p}}_{\text{pool},2}\left(\phi_{1\cap^{\text{​}}2}^{*},\phi_{2\cap^{\text{​}}3}\right){\text{p}}_{2}\left(\phi_{1\cap^{\text{​}}2}^{*},\phi_{2\cap^{\text{​}}3},\psi_{2},Y_{2}\right){\text{p}}_{2}\left(\phi_{1\cap^{\text{​}}2},\phi_{2\cap^{\text{​}}3}\right)}{{\text{p}}_{\text{pool},2}\left(\phi_{1\cap^{\text{​}}2},\phi_{2\cap^{\text{​}}3}\right){\text{p}}_{2}\left(\phi_{1\cap^{\text{​}}2},\phi_{2\cap^{\text{​}}3},\psi_{2},Y_{2}\right){\text{p}}_{2}\left(\phi_{1\cap^{\text{​}}2}^{*},\phi_{2\cap^{\text{​}}3}\right)},$$

(34) $$\alpha \left({\left(\phi_{2\cap^{\text{​}}3}^{*},\psi_{3}^{*}\right)}_{n_{3}^{*}},\left(\phi_{2\cap^{\text{​}}3},\psi_{3}\right)n_{3}\right)=\frac{{\text{p}}_{\text{pool},2}\left(\phi_{1\cap^{\text{​}}2},\phi_{2\cap^{\text{​}}3}^{*}\right){\text{p}}_{2}\left(\phi_{1\cap^{\text{​}}2},\phi_{2\cap^{\text{​}}3}^{*},\psi_{2},Y_{2}\right){\text{p}}_{2}\left(\phi_{1\cap^{\text{​}}2},\phi_{2\cap^{\text{​}}3}\right)}{{\text{p}}_{\text{pool},2}\left(\phi_{1\cap^{\text{​}}2},\phi_{2\cap^{\text{​}}3}\right){\text{p}}_{2}\left(\phi_{1\cap^{\text{​}}2},\phi_{2\cap^{\text{​}}3},\psi_{2},Y_{2}\right){\text{p}}_{2}\left(\phi_{1\cap^{\text{​}}2},\phi_{2\cap^{\text{​}}3}^{*}\right),}$$

(35) $$\alpha \left(\psi_{2}^{*},\psi_{2}\right)=\frac{{\text{p}}_{2}\left(\phi_{1\cap^{\text{​}}2},\phi_{2\cap^{\text{​}}3},\psi_{2}^{*},Y_{2}\right)}{{\text{p}}_{2}\left(\phi_{1\cap^{\text{​}}2},\phi_{2\cap^{\text{​}}3},\psi_{2},Y_{2}\right)}\frac{\text{q}\left(\psi_{2}|\psi_{2}^{*}\right)}{\text{q}\left(\psi_{2}^{*}|\psi_{2}\right)},$$

where *α*(*x, z*) denotes the probability associated with a move from *z* to *x*. Note that all stage two acceptance probabilities only contain terms from the second submodel and the pooled prior, and thus do not depend on *ψ*_1_ or *ψ*_3_. If a move is accepted then we also store the index, i.e. $n_{1}^{*}$ or $n_{3}^{*}$, associated with the move, otherwise we store the current value of the index. The stored indices are used to appropriately resample *ψ*_1_ and *ψ*_3_ from the stage one samples.

### 3.2 Normal approximations to submodel components

Normal approximations are commonly employed to summarise submodels for subsequent use in more complex models. For example, two-stage meta-analyses often use a normal distribution centred on each studies’ effect estimate (Burke et al., 2017). Suppose we employ such an approximation to summarise the prior and posterior of *ϕ*_1∩2_ and *ϕ*_2∩3_ under p_1_ and p_3_ respectively. In addition, assume that (a) such approximations are appropriate for p_1_(*ϕ*_1∩2_), p_1_(*ϕ*_1∩2_ *| Y*_1_), p_3_(*ϕ*_2∩3_), and p_3_(*ϕ*_2∩3_ *| Y*_3_), (b) we are not interested in *ψ*_1_ and *ψ*_3_, and can integrate them out of all relevant densities, and (c) we employ our second form of dictatorial pooling and choose p_2_(*ϕ*_1∩2_, *ϕ*_2∩3_) as the authoritative prior. The latter two assumptions imply that the melded posterior of interest is proportional to

(36) $${\text{p}}_{\text{meld}}\left(\phi_{1\cap^{\text{​}}2},\phi_{2\cap^{\text{​}}3},\psi_{2}|Y\right)\propto \frac{{\text{p}}_{1}\left(\phi_{1\cap^{\text{​}}2}|Y_{1}\right)}{{\text{p}}_{1}\left(\phi_{1\cap^{\text{​}}2}\right)}{\text{p}}_{2}\left(\phi_{1\cap^{\text{​}}2},\phi_{2\cap^{\text{​}}3},\psi_{2}|Y_{2}\right)\frac{{\text{p}}_{3}\left(\phi_{2\cap^{\text{​}}3}|Y_{3}\right)}{{\text{p}}_{3}\left(\phi_{2\cap^{\text{​}}3}\right)}.$$

Denote the normal approximation of p_1_(*ϕ*_1∩2_ | *Y*_1_) as ${\hat{\text{P}}}_{1}\left(\phi_{1\cap^{\text{​}}2}|{\hat{\mu }}_{1},{\hat{\sum }}_{1}\right)$ which is a normal distribution with mean ${\hat{\mu }}_{1}$ and covariance matrix ${\hat{\sum }}_{1}$. The corresponding normal approximation of the prior p_1_(*ϕ*_1∩2_) is ${\hat{\text{P}}}_{1}\left(\phi_{1\cap^{\text{​}}2}|{\hat{\mu }}_{1,0},{\hat{\sum }}_{1,0}\right)$. The equivalent approximations for the subposterior and prior of p_3_ are ${\hat{\text{P}}}_{3}\left(\phi_{2\cap^{\text{​}}3}|{\hat{\mu }}_{3},{\hat{\sum }}_{3}\right)$ and ${\hat{\text{p}}}_{3}\left(\phi_{2\cap^{\text{​}}3}|{\hat{\mu }}_{3,0},{\hat{\sum }}_{3,0}\right)$ respectively. Substituting in the approximations and using standard results for Gaussian density functions (see Bromiley (2003) and Appendix C) results in

(37) $${\hat{\text{p}}}_{\text{meld}}\left(\phi_{1\cap^{\text{​}}2},\phi_{2\cap^{\text{​}}3},\psi_{2}|Y\right)\propto \hat{\text{p}}\left(\left(\phi_{1\cap^{\text{​}}2},\phi_{2\cap^{\text{​}}3}\right)|\hat{\mu },\hat{\sum }\right){\text{p}}_{2}\left(\phi_{1\cap^{\text{​}}2},\phi_{2\cap^{\text{​}}3},\psi_{2}|Y_{2}\right),$$

where

(38) $$\begin{array}{l} {\hat{\mu }}_{\text{nu}}=\left[\begin{array}{l} {\hat{\mu }}_{1}\\ {\hat{\mu }}_{3} \end{array}\right],\;{\hat{\sum }}_{\text{nu}}=\left[\begin{array}{cc} {\hat{\sum }}_{1} & 0\\ 0 & {\hat{\sum }}_{3} \end{array}\right],\;{\hat{\mu }}_{\text{de}}=\left[\begin{array}{c} {\hat{\mu }}_{1,0}\\ {\hat{\mu }}_{3,0} \end{array}\right],\;{\hat{\sum }}_{\text{de}}=\left[\begin{array}{cc} {\hat{\sum }}_{1,0} & 0\\ 0 & {\hat{\sum }}_{3,0} \end{array}\right],\\ \;\hat{\sum }={\left({\hat{\sum }}_{\text{nu}}^{-1}-{\hat{\sum }}_{\text{de}}^{-1}\right)}^{-1},\;\hat{\mu }=\hat{\sum }\left({\hat{\sum }}_{\text{nu}}^{-1}{\hat{\mu }}_{\text{nu}}-{\hat{\sum }}_{\text{de}}^{-1}{\hat{\mu }}_{\text{de}}\right). \end{array}$$

Standard MCMC methods can be used to sample from the approximate melded posterior. If instead we opt for product-of-experts pooling, all ${\hat{\mu }}_{\text{de}}$ and ${\hat{\sum }}_{\text{de}}$ terms disappear from the parameter definitions in Equation (38).

## 4 An integrated population model for little owls

We now return to the integrated population model (IPM) for the little owls introduced in Section 1.1. Finke et al. (2019) consider a number of variations on the original model of Schaub et al. (2006) and Abadi et al. (2010): here we consider only the variant from Finke et al. (2019) with the highest marginal likelihood (Model 4 of their online supplement). This example is particularly interesting to us as, for a certain choice of pooling function and pooling weights, the chained Markov melded model and the IPM are identical. This coincidence allows us to use the posterior from the IPM as a benchmark for our multi-stage sampler.

Before we detail the specifics of each submodel, we must introduce some notation. Data and parameters are stratified into two age-groups *a* ∈ {*J*, *A*} where *J* denotes juvenile owls (less than one year old) and *A* adults, two sexes *s* ∈ {*M*, *F*}, and observations occur annually at times *t* ∈ {1, …, *T* }, with *T* = 25. The sex- and age-specific probability of an owl surviving from time *t* to *t* + 1 is *δ_a,s,t_*, and the sex-specific probability of a previously captured owl being recaptured at time *t* + 1 is *π_s,t_*_+1_ so long as the owl is alive at time *t* + 1.

### 4.1 Capture recapture: p_1_

Capture-recapture data pertain to owls that are released at time *t* (having been captured and tagged), and then recaptured at time *u* = *t* + 1, …, *T*, or not recaptured before the conclusion of the study, in which case *u* = *T* + 1. Define *M_a,s,t,u_* as the number of owls of age-group *a* and sex *s* released at time *t* and recaptured at time *u*. We aggregate these observations into age- and sex-specific matrices ***M**_a,s_*, with *T* rows, corresponding to release times, and *T* +1 columns, corresponding to recapture times. Let $R_{a,s,t}=\sum_{u=1}^{T+1}M_{a,s,t,u}$ be the number of owls released at time *t*, i.e. a vector containing the row-wise sum of the entries in ***M**_a,s_*. The recapture times for owls released at time *t* follow an age- and sex-specific multinomial likelihood

(39) $$\left(M_{a,s,t,1},\ldots ,M_{a,s,t,T+1}\right)∼\text{Multinomial}\left(R_{a,s,t},Q_{a,s,t}\right),$$

with probabilities ***Q**_a,s,t_* = (*Q_a,s,t,_*_1_, …, *Q_a,s,t,T_*_+1_) such that

(40) $$Q_{a,s,t,u}=\{\begin{array}{ll} 0, & \text{for}\;u=1,\ldots ,t\\ \delta_{a,s,t}\pi_{s,u}\prod_{r=t+1}^{u-1}\delta_{a,s,r}\left(1-\pi_{s,r}\right), & \text{for}\;u=t+1,\ldots ,T\\ 1-\sum_{r=1}^{T}\;Q_{a,s,t,r,} & \text{if}\;u=T+1. \end{array}$$

### 4.2 Count data model: p_2_

To estimate population abundance, a two level model is used: the first level models the observed (counted) number of females at each point in time denoted *y_t_*, with a second, latent process modelling the total number of females in population. The observation model is

(41) $$y_{t}|x_{t}∼\text{Poisson}\left(x_{t}\right),$$

where we denote the number of juvenile and adult females in the population at time *t* as *x_J,t_* and *x_A,t_* respectively, with *x_t_* = *x_J,t_* + *x_A,t_*. If sur_*t*_ adult females survive from time *t* − 1 to time *t*, and imm_*t*_ adult females immigrate over the same time period, then the latent, population level model is

(42) $$\begin{array}{c} x_{J,t}|x_{t-1},\rho ,\delta_{J,F,t-1}∼\text{Poisson}\left(x_{t-1}\frac{\rho }{2}\delta_{J,F,t-1}\right),\\ \text{su}{\text{r}}_{t}|x_{t-1},\delta_{A,F,t-1}∼\text{Binomial}\left(x_{t-1},\delta_{A,F,t-1}\right),\\ \text{im}{\text{m}}_{t}|x_{t-1},\eta_{t}∼\text{Poisson}\left(x_{t-1}\eta_{t}\right),\\ x_{A,t}=\text{su}{\text{r}}_{t}+{\text{imm}}_{t}, \end{array}$$

where *η_t_* is the immigration rate. The initial population sizes *x_J,_*_1_ and *x_A,_*_1_ have independent discrete uniform priors on {0, 1, …, 50}. If *x_t−_*_1_ = 0 then we assume that the Poisson and binomial distributions become point masses at zero.

### 4.3 Fecundity: p_3_

The fecundity submodel considers the number of breeding females at time *t* denoted *N_t_*, and the number of chicks produced that survive and leave the nest denoted *n_t_*. A Poisson model is employed to estimate fecundity (reproductive rate) *ρ*

(43) $$n_{t}∼\text{Poisson}\left(N_{t}\rho \right).$$

### 4.4 Parameterisation and melding quantities

Abadi et al. (2010) parameterise the time dependent quantities via linear predictors to minimise the number of parameters in the submodels. The specific parameterisation of Finke et al. (2019) we employ is

(44) $$\begin{array}{c} \text{logit}\left(\delta_{a,s,t}\right)=\alpha_{0}+\alpha_{1}𝕀(s=M)+\alpha_{2}𝕀(a=A),\;\text{log}\left(\eta_{t}\right)=\alpha_{6},\\ \text{logit}\left(\pi_{s,u}\right)=\alpha_{4}𝕀(s=M)+\alpha_{5,u},\;\text{for}\;u=2,\ldots T, \end{array}$$

thus the quantities in common between the submodels are *ϕ*_1∩2_ = (*α*_0_, *α*_2_) and *ϕ*_2∩3_ = *ρ*. To align the notation of this example with our chained melding notation we define, for all permitted values of *a, s* and *t*, *Y*_1_ = (*M_a,s_*), $\psi_{1}=\left(\alpha_{1},\alpha_{4},{\left(\alpha_{5,u}\right)}_{u=2}^{T}\right)$ *Y*_2_ = (*y_t_*), *ψ*_2_ = (*x_J,t_, α*_6_, sur_*t*_, imm_*t*_); and *Y*_3_ = (*N_t_, n_t_*), *ψ*;_3_ = ∅. Note that the definition of *ϕ*_1∩2_ does not include *α*_1_ as it is male specific and does not exist in p_2_. The model variant of Finke et al. (2019) we consider does not include *α*_3_, and for comparability we keep the other parameter indices the same.

### 4.5 Priors

We use the priors of Finke et al. (2019) for the parameters in each submodel. Denote *α* = (*α*_0_, *α*_1_, *α*_2_, *α*_4_, *α*_6_). In both p_1_ and p_2_ the elements of *α* are assigned independent Normal(0, 2^2^) priors truncated to [−10, 10]. The time varying recapture probabilities *α*_5,*u*_ also have Normal(0, 2^2^) priors truncated to [−10, 10]. A Uniform(0, 10) prior is assigned to *ρ* in p_2_ and p_3_.

To completely specify p_meld_ we must choose how to form p_pool_(*ϕ*_1∩2_, *ϕ*_2∩3_). We form p_pool_(*ϕ*_1∩2_, *ϕ*_2∩3_) using three different pooling methods and estimate the melded posterior in each case. The first pooling method is product-of-experts (PoE) pooling, which is logarithmic pooling with *λ* = (1, 1, 1), and we denote the melded posterior as p_meld,PoE_. We also use logarithmic pooling with $\lambda =\left(\frac{1}{2},\frac{1}{2},\frac{1}{2}\right)$, which is denoted p_meld,log_ and results in the chained melded model being identical to the IPM. The final pooling method is linear pooling with $\lambda =\left(\frac{1}{2},\frac{1}{2},\frac{1}{2},\frac{1}{2}\right)$, denoted p_meld,lin_.

### 4.6 Posterior estimation

We estimate the melded posterior – p_meld_(*ϕ, ψ | Y*), proportional to Equation (9) – using both the parallel sampler (Section 3.1) and normal approximation (Section 3.2). This allows us to use pre-existing implementations of the submodels. Specifically, the capture-recapture submodel is written in BUGS (Lunn et al., 2009) and sampled via rjags (Plummer, 2019). The fecundity submodel is written in Stan (Carpenter et al., 2017) and sampled via rstan (Stan Development Team, 2021). The count data submodel is also written in BUGS, and we reuse this implementation in stage two of the multi-stage sampler via NIMBLE (de Valpine et al., 2017) and its R interface (NIMBLE Development Team, 2019). The approximate melded posterior obtained by Section 3.2 is sampled using rjags. Code and data for this example, as well as trace plots and numerical convergence measures (Vehtari et al., 2020) for both stages of the parallel sampling process, are available in the accompanying online repository^5^.

### 4.7 Results

**Figure 5:**
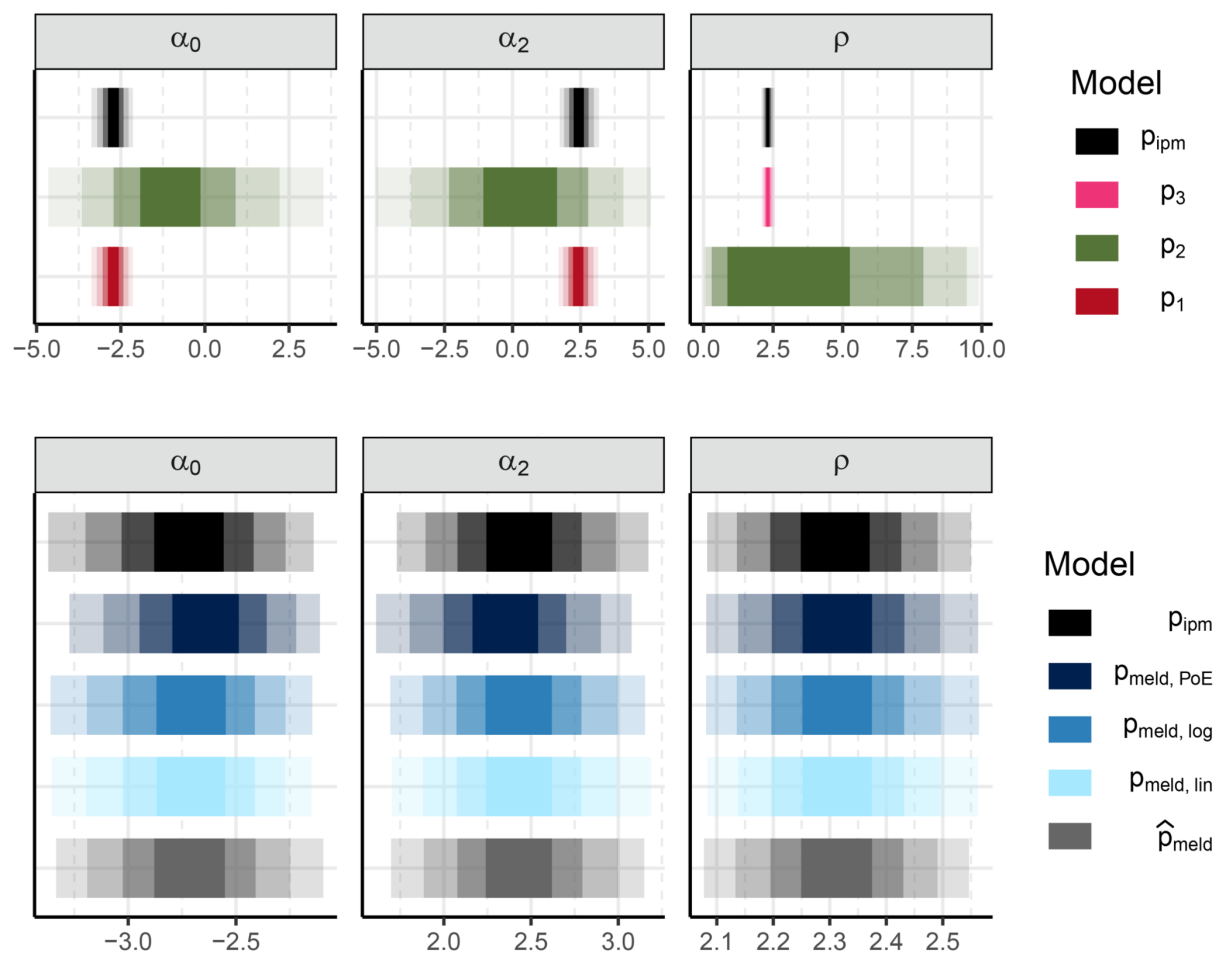
Top row: credible intervals for *ϕ*_1∩2_ = (*α*_0_, *α*_2_) and *ϕ*_2∩3_ = *ρ* from the posterior of the original integrated population model p_ipm_, and the individual subposteriors from submodels p_1_, p_2_, and p_3_. Bottom row: credible intervals for the same quantities, but with a different x-axis scale, from the original IPM (repeated from top row); the chained melded posteriors using product-of-experts pooling, logarithmic pooling, and linear pooling denoted p_meld_, p_meld,log_ and p_meld,lin_; and the melded posterior using the normal approximation ${\hat{\text{P}}}_{\text{meld}}$. Intervals are 50%, 80%, 95%, and 99% wide.

We empirically validate our methodology and sampler by comparing the melded posterior samples to a large sample – 6 chains, each containing 1×10^5^ post-warmup iterations – from the original IPM posterior. Similarity in the posteriors is expected as the IPM is effectively the joint model we wish to approximate with the chained melded model. It is simply fortunate, from a modelling standpoint, that this example’s joint model is easy to construct and computationally feasible with standard tools. Note that under logarithmic pooling with $\lambda =\left(\frac{1}{2},\frac{1}{2},\frac{1}{2}\right)$ the melded posterior is identical to the original IPM, so any differences between the two posteriors are attributable to the multi-stage sampler. Figure 5 depicts the posterior credible intervals (Gabry et al., 2021; Kay, 2020) for the common quantities from the individual submodels, the melded models, and the original IPM. The top row in Figure 5 indicates that the count data alone (p_2_) contain minimal information about *α*_0_, *α*_2_ and *ρ*; incorporating the data from the other submodels is essential for precise estimates.

The multi-stage sampler works well by producing melded posterior estimates generally similar to the original IPM estimate, and are near identical for logarithmic pooling. PoE pooling produces the posterior most different from the original IPM, as it yields a prior for (*α*_0_, *α*_2_) that is more concentrated around zero than the other pooling methods. The lack of large differences between the melded posteriors that use different pooled priors indicates that the prior has almost no effect on the posterior. The similarity of the approximate approach (${\hat{\text{p}}}_{\text{meld}}$ - bottom row of Figure 5) to the melding approaches suggests that the normal approximations are good summaries of the subposteriors, and that the approximate melding procedure of Section 3.2 is suitable for this example.

## 5 Survival analysis with time varying covariates and uncertain event times

We return now to the respiratory failure example introduced in Section 1.1. Our intention is to illustrate the application of chained Markov melding to an example of realistic complexity, and explore empirically the importance of accounting for all sources of uncertainty by comparing chained Markov melding to equivalent analyses which use only a point estimate summary of the uncertainty. Specifically, event times and indicators are a noninvertible function of other parameters in the first submodel, and are an uncertain response in the survival submodel. Chained Markov melding enables us to specify a suitable joint model despite these complications.

There are *i* = 1, …, *N* individuals in the data set. Each individual is admitted to the ICU at time 0, and is discharged or dies at time *C_i_*. See Appendix I for information on how the *N* = 37 individuals were selected from MIMIC-III (Johnson et al., 2016).

### 5.1 P/F ratio submodel (B-spline): p_1_

The first submodel fits a B-spline to the PaO_2_/FiO_2_ data to calculate if and when an individual experiences respiratory failure. Each individual has PaO_2_/FiO_2_ ratio observations *z_i,j_* (in units of mmHg) at times *t_i,j_*, with *j* = 1, …, *J_i_*. For each individual denote the vector of observations *z_i_* = (*z_i,_*_1_, …, *z_i,Ji_*) and observation times *t_i_* = (*t_i,_*_1_, …, *t_i,Ji_*). To improve computational performance, we standardise the P/F ratio data for each individual such that $z_{i,j}=\frac{{\tilde{z}}_{i,j}-{\bar{z}}_{i}}{{\hat{s}}_{i}}$, where ${\tilde{z}}_{i,j}$ is the underlying unstandardised observation with mean ${\bar{z}}_{i}$ and standard deviation ${\hat{s}}_{i}$. Similarly we rescale the threshold for respiratory failure: $\tau_{i}=\frac{300-{\bar{z}}_{i}}{{\hat{s}}_{i}}$.

We choose to model the P/F ratio using cubic B-splines and 7 internal knots, and do not include an intercept column in the spline basis (for background on B-splines see: Chapter 2 in Hastie and Tibshirani, 1999; and the supplementary material of Wang and Yan, 2021). The internal knots are evenly spaced between two additional boundary knots at min(*t_i_*) and max(*t_i_*). These choices result in *k* = 1, …, 10 spline basis terms per individual, with coefficients *ζ_i,k_* where *ζ_i_* = (*ζ_i,_*_1_, …, *ζ_i,_*_10_). We denote the individual specific B-spline basis evaluated at time *t_i,j_* as *B_i_*(*t_i,j_*) *∈* [0, *∞*)^10^ so that the submodel can be written as

(45) $$z_{i,j}=\beta_{0,i}+B_{i}{\left(t_{i,j}\right)}^{⊤}\zeta_{i}+\varepsilon_{i,j}.$$

We employ a weakly informative prior for the intercept *β*_0,*i*_ ∼ N(0, 1^2^), a heavy tailed distribution for the error term^6^ *ε_i,j_ ∼ t*_5_(0, *ω_i_*), and a weakly informative half-normal prior for the unknown scale parameter *ω_i_ ∼* N_+_(0, 1^2^). For the spline basis coefficients we set *ζ_i,_*_1_ *∼* N(0, 0.1^2^), and for *k* = 2, …, 10 we employ the random-walk prior *ζ_i,k_ ∼* N(*ζ_i,k−_*_1_, 0.1^2^) from Kharratzadeh (2017).

We identify that a respiratory failure event occurred (which we denote by *d_i_* = 1) at event time *T_i_* if a solution to the following optimisation problem exists

(46) $$T_{i}=\underset{t}{\text{min}}\;\left\{\tau_{i}=\beta_{0,i}+B_{i}(t)\zeta_{i}|t\in \left[\text{max}\left(0,\text{min}\left({\text{t}}_{i}\right)\right),\;\text{max}\left({\text{t}}_{i}\right)\right]\right\},$$

**Figure 6:**
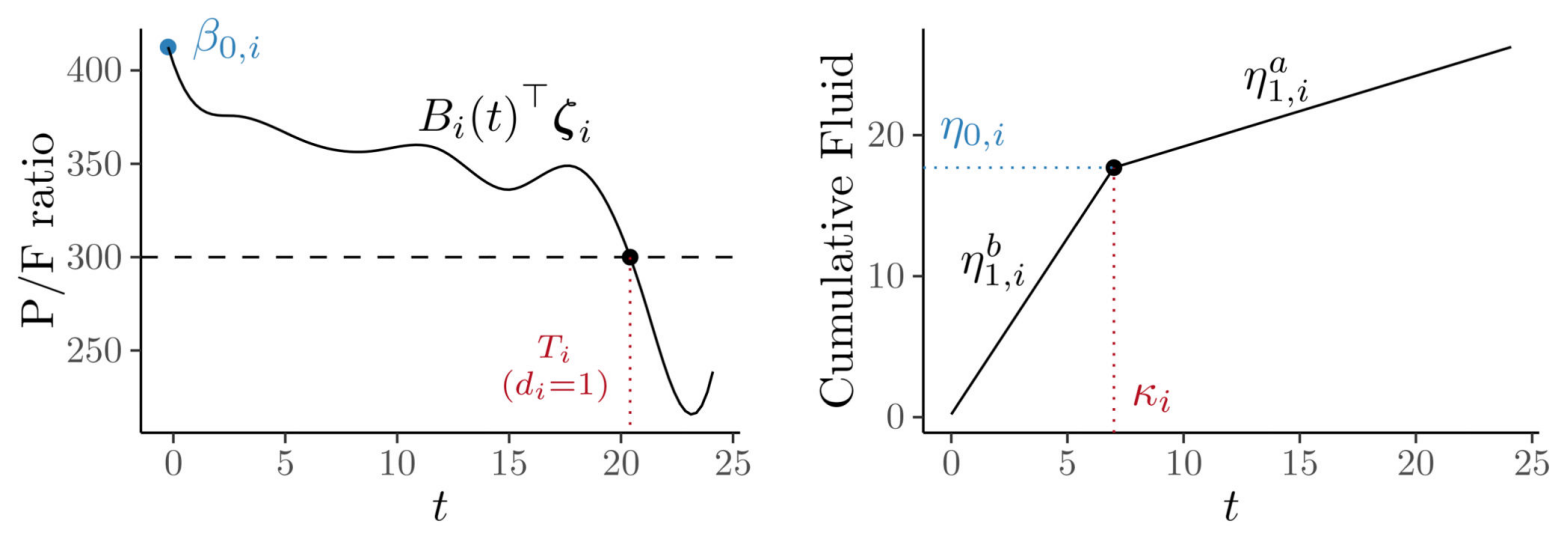
Parameters and form for the P/F ratio submodel (p_1_, left) and cumulative fluid submodel (p_3_, right).

We attempt to solve Equation 46 using a standard multiple root finder (Soetaert et al., 2020). If there are no roots then the individual died or was discharged before respiratory failure occurred so we set *T_i_* = *C_i_* and *d_i_* = 0. The relationship between *T_i_* and other model coefficients is displayed in the left hand panel of Figure 6.

### 5.2 Cumulative fluid submodel (piecewise linear) p_3_

The rate of fluid administration reflects the clinical management of patients by ICU staff, and hence changes to the rate reflect decisions to change treatment strategy. We employ a breakpoint regression model to capture the effect of such decisions, and consider only one breakpoint as this appears sufficient to fit the observed data. Specifically, we model the 8-hourly cumulative fluid balance data *x_i,l_* (in litres) at times *u_i,l_*, *l* = 1, …, *L_i_*.

The cumulative data are derived from the raw fluid input/output observations, which we detail in Appendix D. We denote the complete vector of observations by *x_i_* = (*x_i,_*_1_, …, *x_i,Li_*) and times by *u_i_* = (*u_i,_*_1_, …, *u_i,Li_*).

We assume a piecewise linear model with *η*_0,*i*_ as the value at the breakpoint at time *κ_i_*, slope $\eta_{1,i}^{b}$ before the breakpoint, and slope $\eta_{1,i}^{a}$ after the breakpoint. We write this submodel as

(47) $$\begin{array}{c} x_{i,l}=m_{i}\left(u_{i,l}\right)+\epsilon_{i,l},\\ m_{i}\left(u_{i,l}\right)=\eta_{0,i}+\eta_{1,i}^{b}\left(u_{i,l}-\kappa_{i}\right){𝟙}_{\left\{u_{i,l}<\kappa_{i}\right\}}+\eta_{1,i}^{a}\left(u_{i,l}-\kappa_{i}\right){𝟙}_{\left\{u_{i,l}\geq \kappa_{i}\right\}}. \end{array}$$

It will be useful to refer to the fitted value of this submodel at arbitrary time as *m_i_*(*t*). We assume a weakly informative prior for the error term $\epsilon_{i,l}∼\text{N}\left(0,\sigma_{x,i}^{2}\right)$, with individual-specific error variances *σ_x,i_ ∼* N_+_(0, 5^2^), and specific, informative priors for the slope before the breakpoint $\eta_{1,i}^{b}$ ∼ Gamma(1.53, 0.24) and after $\eta_{1,i}^{a}$ ∼ Gamma(1.53, 0.24). An appropriate prior for *κ_i_* and *η*_0,*i*_ is challenging to specify due to the relationship between the two parameters and the individual-specific support for *κ_i_*. We address both challenges by reparameterisation, resulting in a prior for *κ_i_* that, in the absence of other information, places the breakpoint in the middle of an individual’s ICU stay, and a prior for *η*_0,*i*_ that captures the diverse pathways into ICU that an individual can experience. Details and justifications for all the informative priors are available in Appendix E. Figure 6 displays the parameters and their relationship to the fitted regression line.

### 5.3 Survival submodel p_2_

The rate at which fluid is administered is thought to influence the time to respiratory failure (Seethala et al., 2017), so we explore this relationship using a survival model. Individuals experience respiratory failure (*d_i_* = 1) at time 0 *< t < C_i_*, or are censored (*d_i_* = 0, *t* = *C_i_*). We assume a Weibull hazard with shape parameter *γ* for the event times. All individuals have baseline (time invariant) covariates *w_i,a_*, *a* = 1, …, *A*, with *w_i_* = (1, *w_i,_*_1_, …, *w_i,A_*) (i.e. including an intercept term), and common coefficients *θ* = (*θ*_0_, …, *θ_A_*). The hazard is assumed to be influenced by these covariates and the rate of increase $\frac{\partial }{\partial t}m_{i}(t)$ in the cumulative fluid balance. The strength of the latter relationship is captured by *α*. Hence, the hazard is

(48) $$h_{i}(t)=\gamma t^{\gamma -1}\exp\left\{w_{i}^{⊤}\text{θ}+\alpha \frac{\partial }{\partial t}m_{i}(t)\right\},$$

(49) $$\frac{\partial }{\partial t}m_{i}(t)=\eta_{1,i}^{b}{𝟙}_{\left\{t<\kappa_{i}\right\}}+\eta_{1,i}^{a}{𝟙}_{\left\{t\geq \kappa_{i}\right\}},$$

The survival function at an individual’s observed event time and status, (*T_i_, d_i_*), denoted $S_{i}\left(T_{i}\right)=\exp\left\{-\int_{0}^{T_{i}}h_{i}(u)\text{d}u\right\}$, has an analytic form which we derive in Appendix F.

Thus, the likelihood for individual *i* is

(50) $$\text{p}\left(T_{i},d_{i}|\gamma ,\text{θ},\alpha ,\kappa_{i},\eta_{1,i}^{b},\eta_{1,i}^{a},{\text{w}}_{i}\right)=h_{i}{\left(T_{i}\right)}^{d_{i}}S_{i}\left(T_{i}\right),$$

where we suppress the dependence on the parameters on the right hand side for brevity.

Our priors, which we justify in Appendix G, for the submodel specific parameters are *γ ∼* Gamma(9.05, 8.72), *α ∼* SkewNormal(0, 0.5, −2), *θ_a_ ∼* SkewNormal(0, 0.5, −1), and *θ*_0_ *∼* N(*Ê*, 0.5^2^) where *Ê* is the log of the crude event rate (Brilleman et al., 2020). We adopt the same priors as the cumulative fluid balance submodel for *κ_i_*, $\eta_{1,i}^{b}$, and $\eta_{1,i}^{a}$.

### 5.4 Chained Markov melding details

To combine the submodels with chained Markov melding we must define the common quantities *ϕ*_1∩2_ and *ϕ*_2∩3_. We meld p_1_ and p_2_ by treating the derived event times and indicators ${\left\{\left(T_{i},d_{i}\right)\right\}}_{i=1}^{N}$ under p_1_ as the “response”, i.e. event times, in p_2_. Care is required when defining *ϕ*_1∩2_ under p_1_ as it is a deterministic function of *β*_0, *i*_ and *ζ_i_*. Define *χ*_1,*i*_ = (*β*_0, *i*_, *ζ*_i_) and *ϕ*_1∩2,*i*_ = *f*(*χ*_1,*i*_) = (*T_i_, d_i_*), where *f* is the output from attempting to solve Equation (46), so that *ϕ*_1∩2_ = (*f*(*χ*_1, *i*_), …, *f*(*χ*_1,*N*_)). The parameters shared by Equations (47) and (49) constitute $\phi_{2\cap 3}={\left(\eta_{1,i}^{b},\eta_{1,i}^{a},\kappa_{i}\right)}_{i=1}^{N}$.

To completely align with our chained melding notation we also define, for the P/F submodel, $Y_{1}={\left(z_{i},{\text{t}}_{i}\right)}_{i=1}^{N}$ and $\psi_{1}={\left(\omega_{i}\right)}_{i=1}^{N}$, noting that *ψ*_1_ and (*χ*_1,*i*_, …, *χ*_1,*N*_) have no components in common. For the cumulative fluid submodel we define $Y_{3}={\left({\text{x}}_{i},{\text{u}}_{i}\right)}_{i=1}^{N}$, and $\psi_{3}={\left(\eta_{0,i},\sigma_{x,i}^{2}\right)}_{i=1}^{N}$. Finally, for the survival submodel we define $Y_{2}={\left({\text{w}}_{i}\right)}_{i=1}^{N}$ and *ψ*_2_ = (*γ, **θ**, α*).

### 5.5 Pooling and estimation

We consider logarithmic pooling with $\lambda =\left(\frac{4}{5},\frac{4}{5},\frac{4}{5}\right)$ (any smaller value of *λ* results in a prior that is so uninformative that it causes computational problems) and with *λ* = (1, 1, 1) (Product-of-Experts). Because the correlation between *ϕ*_1∩2_ and *ϕ*_2∩3_ in p_2_(*ϕ*_1∩2_, *ϕ*_2∩3_) is important, we do not consider linear pooling in this example. Logarithmic pooling requires estimates of p_1_(*ϕ*_1∩2_) and p_2_(*ϕ*_1∩2_, *ϕ*_2∩3_). Because these are mixed distributions, with both discrete and continuous components, standard kernel density estimation, as suggested by Goudie et al. (2019), is inappropriate. Instead we fit, to transformed versions of *ϕ*_1∩2_ and *ϕ*_2∩3_, a mixture containing a discrete component and either a Gaussian or beta distribution, depending on the transformation. Further details for all the mixture distribution estimates are contained in Appendix H.

We use the parallel multi-stage sampler with p_pool,1_(*ϕ*_1∩2_) = p_1_(*ϕ*_1∩2_), p_pool,3_(*ϕ*_2∩3_) = p_3_(*ϕ*_2∩3_) and p_pool,2_(*ϕ*_1∩2_, *ϕ*_2∩3_) = p_pool_(*ϕ*) */* (p_1_(*ϕ*_1∩2_)p_3_(*ϕ*_2∩3_)). That is, in stage one we target the subposteriors p_1_(*ϕ*_1∩2_, *ψ*_1_ *| Y*_1_) and p_3_(*ϕ*_2∩3_, *ψ*_3_ *| Y*_3_); in stage two we target the full melded model. Targeting p_1_(*ϕ*_1∩2_, *ψ*_1_ *| Y*_1_) in stage one alleviates the need to solve Equation (46) within an MCMC iteration, instead turning the production of *ϕ*_1∩2_ into an embarrassingly parallel, post-stage-one processing step. Attempting to sample the melded posterior directly would involve solving (46) many times within each iteration, presenting a sizeable computational hurdle which we avoid. It is crucial for the convergence of our multi-stage sampler that the components of *ϕ*_1∩2_ and *ϕ*_2∩3_ are updated *individual-at-a-time* in stage two. This is possible due to the conditional independence between individuals in the stage one posterior, and Appendix K contains the details of this scheme. The stage one subposteriors are sampled using Stan, using 5 chains with 10^3^ warm-up iterations and 10^4^ post warm-up iterations. We use Stan to sample *ψ*_2_ where, in every MH-within-Gibbs step, we run Stan for 9 warm-up iterations and 1 post warm-up iteration^7^. We run 5 chains of 10^4^ iterations for all stage two targets. Visual and numerical diagnostics (Vehtari et al., 2020) are assessed and are available in the repository accompanying this paper^8^.

### 5.6 Results

**Figure 7:**
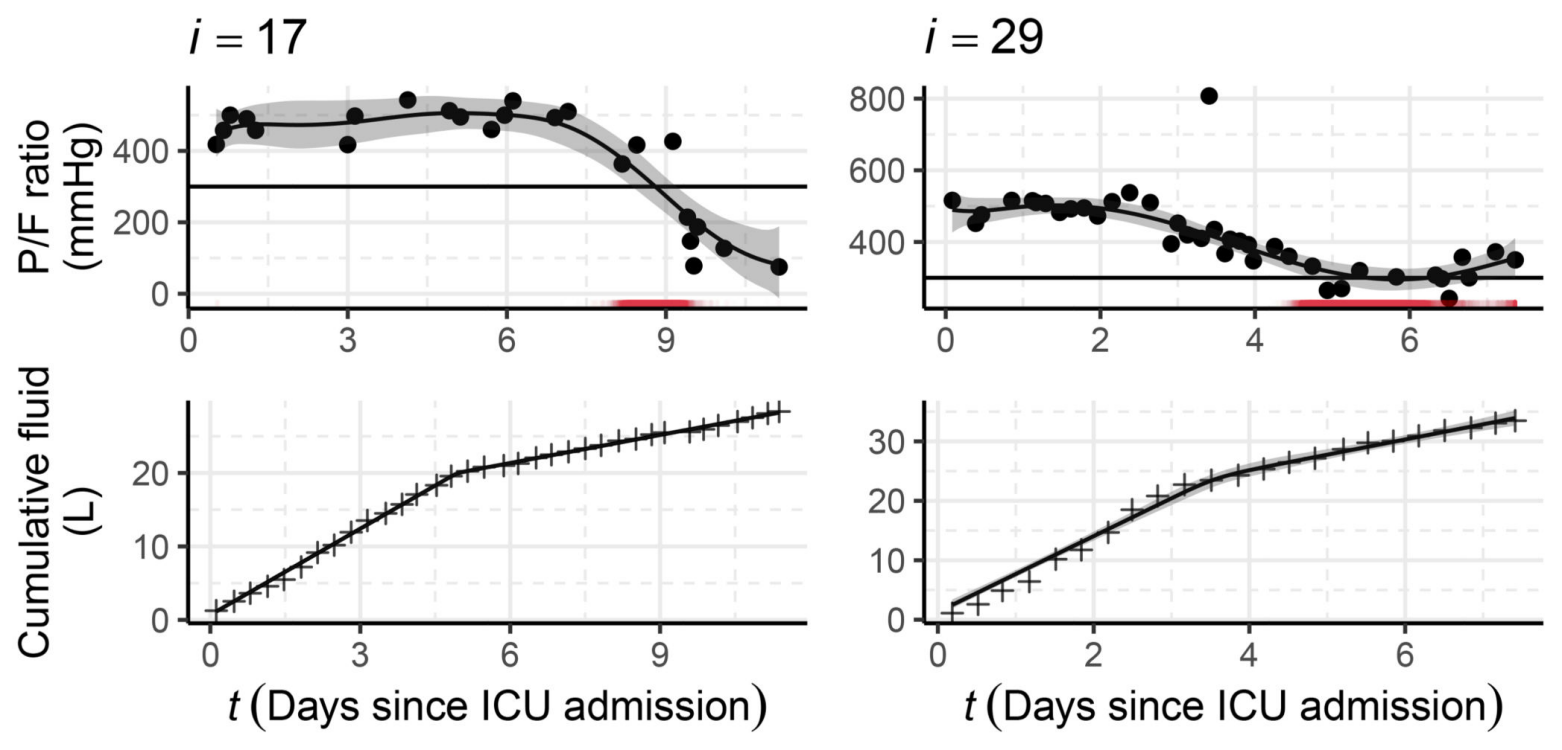
The P/F ratio data (*Y*_1_, top row); cumulative fluid data (*Y*_3_, bottom row); subposterior means and 95% credible intervals for each of the submodels (black solid lines and grey intervals); and stage one event times (*T_i_*, red rug in the top row) for individuals *i* = 17 and 29.

We first inspect the subposterior fitted values for p_1_ and p_3_. The top row of Figure 7 displays the P/F data, the fitted submodel, and derived event times for individuals *i* = 17 and 29. The spline appears to fit the raw P/F data well, with the heavy tailed error term accounting for the larger deviations away from the fitted value. It is interesting to see the relatively wide, multimodal distribution for (*T*_29_, *d*_29_) (there is a second mode at (*T*_29_ = *C*_29_, *d*_29_ = 0) and for other individuals not shown here). The bottom row of Figure 7 displays the cumulative fluid data and the fitted submodel, with the little noise in the data resulting in minimal uncertainty about the fitted value and a concentrated subposterior distribution.

To assess the importance of fully accounting for the uncertainty in *ϕ*_1∩2_ and *ϕ*_2∩3_, we compare the posterior for *ψ*_2_ obtained using the chained melding approach with the posterior obtained by fixing *ϕ*_1∩2_ and *ϕ*_2∩3_. Plugging in a point estimate reflects common applied statistical practice when combining submodels, particularly when a distributional approximation is difficult to obtain (as it is for p_1_(*ϕ*_1∩2_ *| Y*_1_)). Additionally, standard survival models and software typically do not permit uncertainty in event times and indicators, rendering such a plug-in approach necessary.

**Figure 8:**
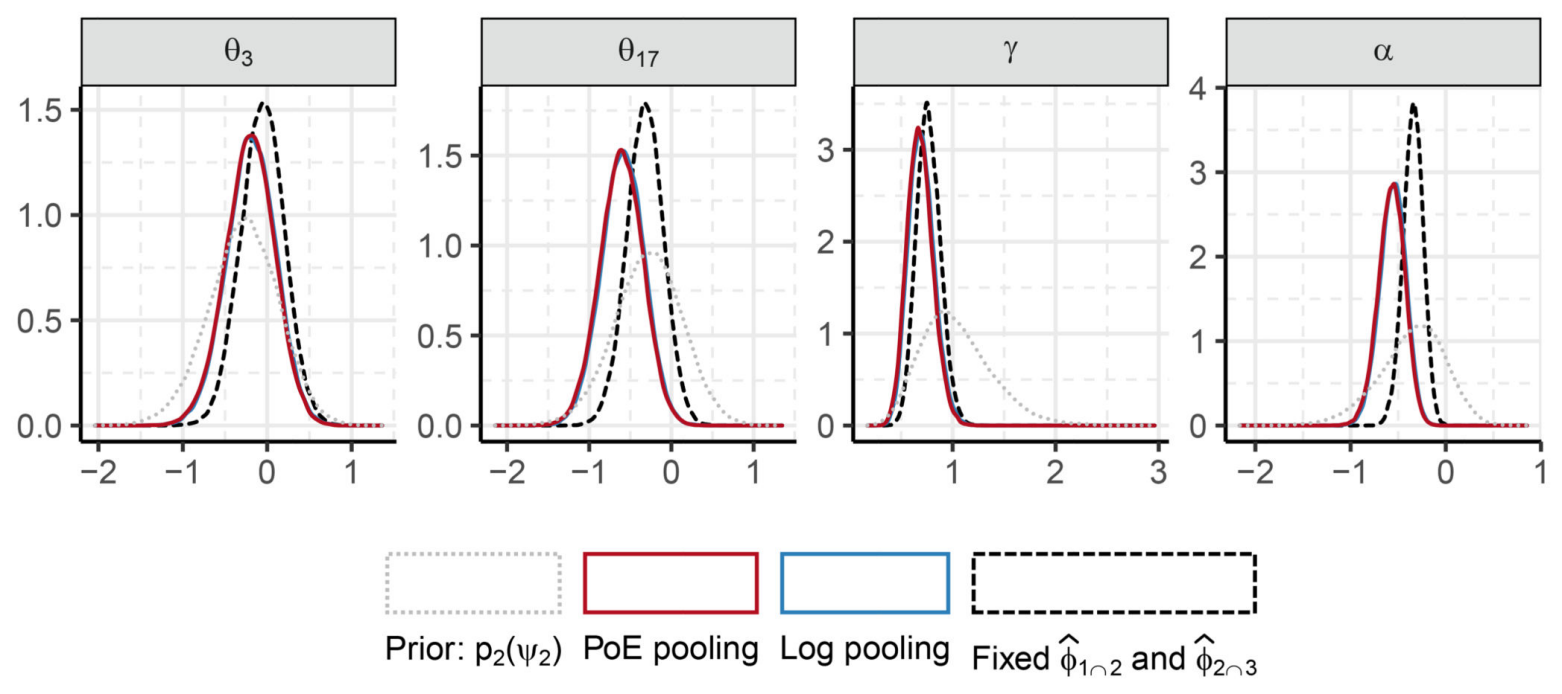
Density estimates for a subset of *ψ*_2_. The submodel marginal prior p_2_(*ψ*_2_) is shown as the grey dotted line (note that this is not the marginal prior under the melded model). The figure also contains the subposteriors obtained from chained melding using PoE pooling (red, solid line) and logarithmic pooling (blue, solid line), as well as the posterior using the fixed values $\text{p}\left(\psi_{2}|{\hat{\phi }}_{1\cap 2},{\hat{\phi }}_{2\cap 3},Y_{2}\right)$ (black, dashed line).

Specifically, we fix *ϕ*_1∩2_ to the median value^9^ for each individual under p_1_(*ϕ*_1∩2_ *| Y*_1_) and denote it ${\hat{\phi }}_{1\cap 2}$ and use the subposterior mean of p_3_(*ϕ*_2∩3_ *| Y*_3_) denoted ${\hat{\phi }}_{2\cap 3}$ With these fixed values we sample $\text{p}\left(\psi_{2}|{\hat{\phi }}_{1\cap 2},{\hat{\phi }}_{2\cap 3},Y_{2}\right)$. We also compare the melded posterior to the submodel marginal prior p_2_(*ψ*_2_), but we note that this comparison is difficult to interpret, as the melding process alters the prior for *ψ*_2_. Figure 8 displays the aforementioned densities for (*θ*_3_, *θ*_17_, *γ, α*) *⊂ ψ*_2_, with (*θ*_3_, *θ*_17_) chosen as they exhibit the greatest sensitivity to the fixing of *ϕ*_1∩2_ and *ϕ*_2∩3_. For the baseline coefficients (*θ*_3_, *θ*_17_) the chained melding posterior differs slightly in location from $\text{p}\left(\psi_{2}|{\hat{\phi }}_{1\cap 2},{\hat{\phi }}_{2\cap 3},Y_{2}\right)$ with a small increase in uncertainty. A more pronounced change is visible for *α*, where the melding process has added a notable degree of uncertainty and shifted the posterior leftwards.

**Figure 9:**
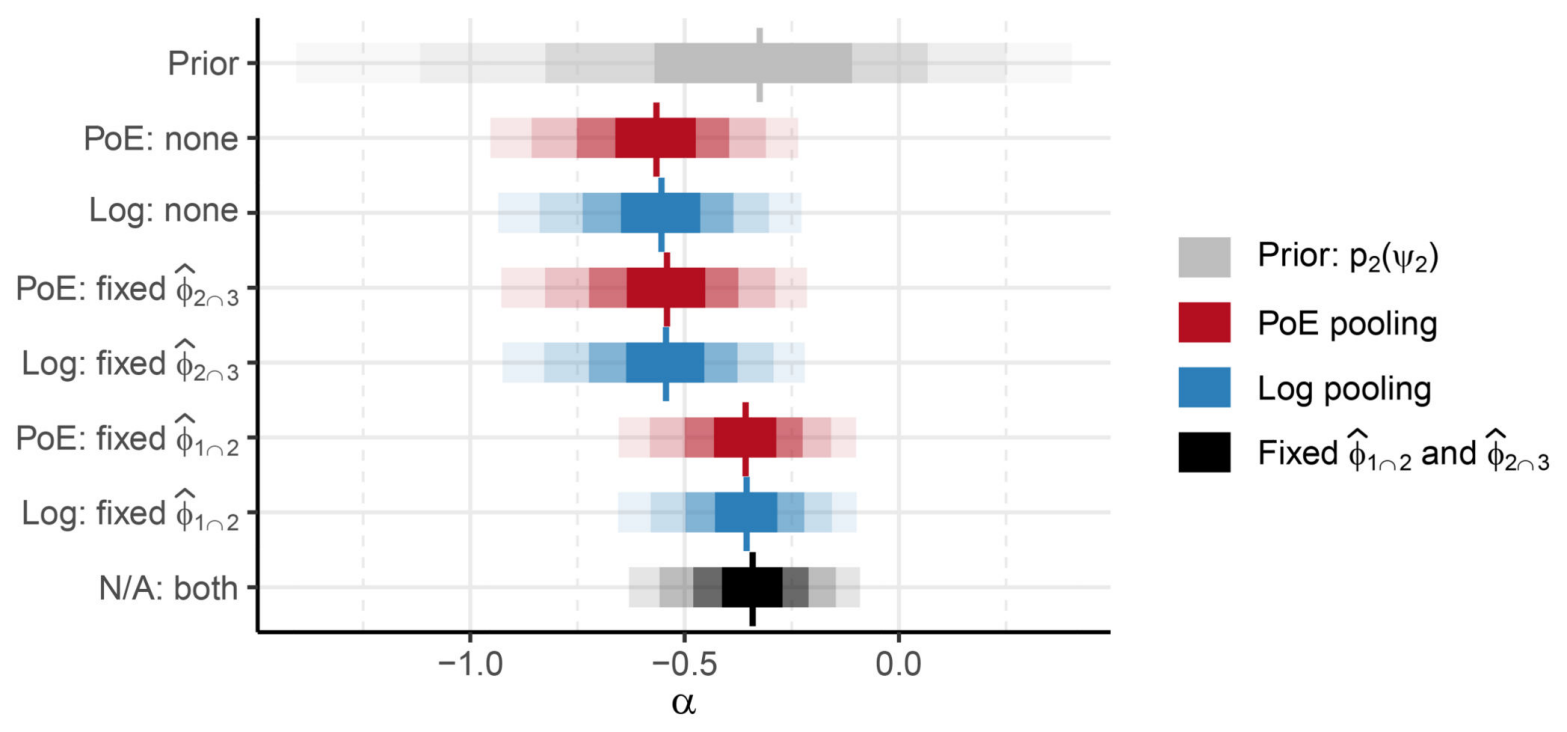
Median (vertical line), 50%, 80%, 95%, and 99% credible intervals (least transparent to most transparent) for *α*. The marginal prior (grey, top row) and posterior using fixed ${\hat{\phi }}_{1\cap 2}$ and ${\hat{\phi }}_{2\cap 3}$ (black, bottom row) are as in Figure 8. For the chained melded posteriors (red and blue, rows 2 and 3) and the melded posteriors (red and blue, rows 4 – 7), the tick label on the y-axis denotes the type of pooling used, and which of *ϕ*_1∩2_ and/or *ϕ*_2∩3_ are fixed.

To investigate which part of the melding process causes this change in the posterior of *α*, we consider fixing either one of *ϕ*_1∩2_ and *ϕ*_2∩3_ to their respective point estimates. That is, we employ Markov melding as described in Section 1.2, using either logarithmic or PoE pooling, to obtain ${\text{p}}_{\text{meld}}\left(\alpha |{\hat{\phi }}_{1\cap 2},Y_{2},Y_{3}\right)$ and ${\text{p}}_{\text{meld}}\left(\alpha |{\hat{\phi }}_{2\cap 3},Y_{1},Y_{2}\right)$. Figure 9 displays the same distributions for *α* as Figure 8, and adds the posteriors obtained using one fixed value $\left({\hat{\phi }}_{1\cap 2}\text{or}{\hat{\phi }}_{2\cap 3}\right)$ whilst melding the other non-fixed parameter.

Evident for both choices of pooling is the importance of incorporating the uncertainty in *ϕ*_1∩2_. This is expected given the large uncertainty and multimodal nature of *ϕ*_1∩2_ compared to *ϕ*_2∩3_ (see Figure 7). We suspect that it is the multimodality in p_1_(*ϕ*_1∩2_ *| Y*_1_) that produces the shift in posterior mode of *ϕ*_1∩2_, with the width of p_1_(*ϕ*_1∩2_ *| Y*_1_) affecting the increase in uncertainty. Because we prefer the chained melded posterior, under either pooling method, for its full accounting of uncertainty we conclude that $\text{p}\left(\alpha |{\hat{\phi }}_{1\cap 2},{\hat{\phi }}_{2\cap 3},Y_{2}\right)$ is both overconfident and biased.

**Figure 10:**
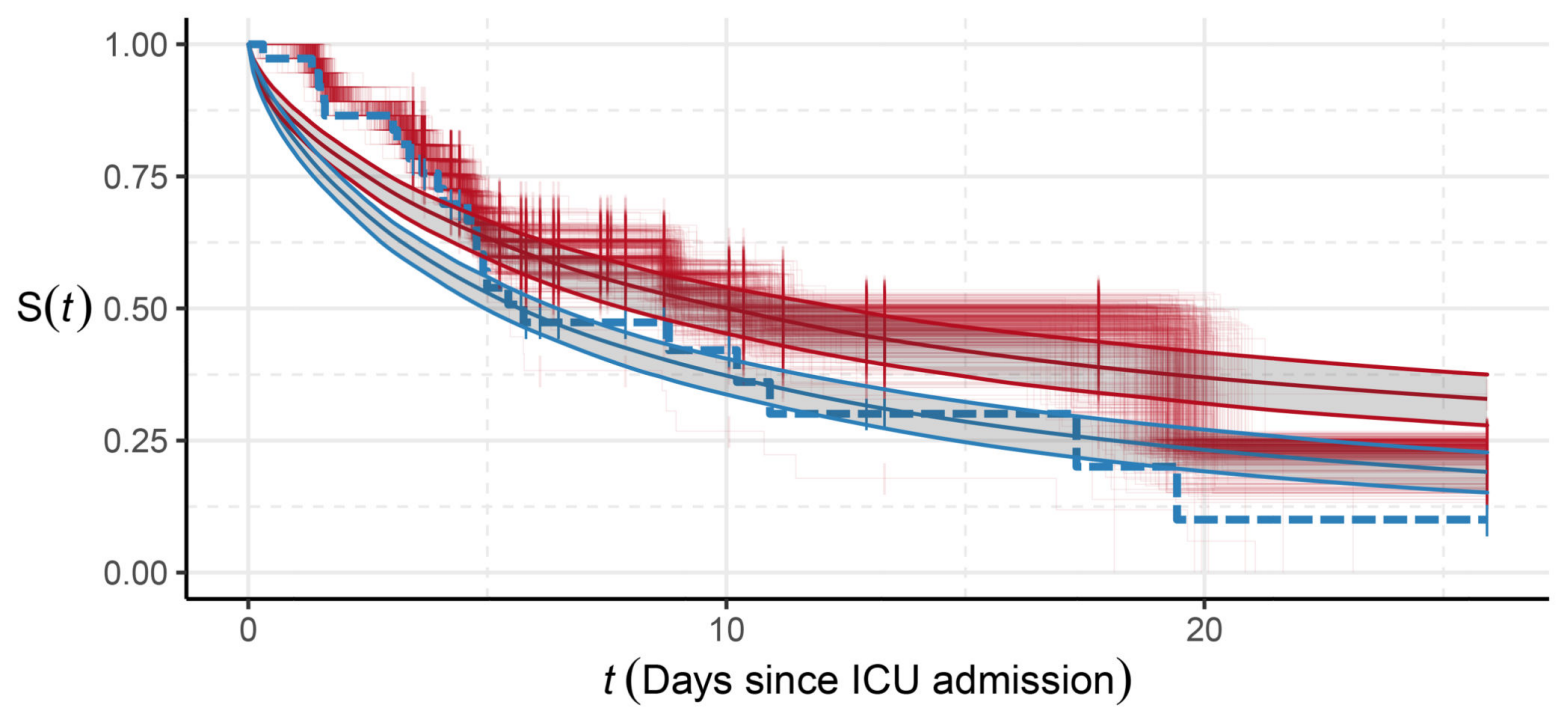
Survival curves and mean survival function at time *t*. The red, stepped lines are draws of *ϕ*_1∩2_ from the melded posterior using PoE pooling, converted into survival curves via the Kaplan-Meier estimator. The smooth red line and interval (posterior mean and 95% credible interval) denote the model-based, mean survival function obtained from the melded posterior (PoE pooling) values of *ψ*_2_ and *ϕ*_2∩3_. The blue dashed line is the Kaplan-Meier estimate of ${\hat{\phi }}_{1\cap 2}$, and the blue solid line and interval are the corresponding model-based estimate from $\text{p}\left(\psi_{2}|{\hat{\phi }}_{1\cap 2},{\hat{\phi }}_{2\cap 3},Y_{2}\right)$.

The marginal changes to the components of *ψ*_2_ visible in Figure 8 appear small, however the cumulative effect of such changes becomes apparent when inspecting the posterior of the survival function. Figure 10 displays the model-based, mean survival function under the melded posterior (using PoE pooling), and corresponding draws of *ϕ*_1∩2_ converted into survival curves using the Kaplan-Meier estimator. Also shown are the Kaplan-Meier estimate of ${\hat{\phi }}_{1\cap 2}$ and the mean survival function computed using $\text{p}\left(\psi_{2}|{\hat{\phi }}_{1\cap 2},{\hat{\phi }}_{2\cap 3},Y_{2}\right)$. The posterior survival functions differ markedly, with the 95% intervals overlapping only for small values of time. It is also interesting to see that ${\hat{\phi }}_{1\cap 2}$, despite being a reasonable point estimate of p_1_(*ϕ*_1∩2_ *| Y*_1_), is not very likely under the melded posterior. Figure 10 also suggests that the Weibull hazard is insufficiently flexible for this example. We discuss the complexities of other hazards in Section 6.

## 6 Conclusion

This paper introduces the chained Markov melded model. In doing so we make explicit the notion of submodels related in a chain-like way, describe a generic methodology for joining together any number of such submodels and illustrate its application with our examples. Our examples also demonstrate the importance of quantifying the uncertainty when joining submodels; not doing so can produce biased, over-confident inference. We also present the choices, and their impacts, that users of chained Markov melding must make which include: the choice of pooling function, and where required the pooling weights; the choice of posterior sampler and the design thereof, including the apportionment of the pooled prior over the stages and stage-specific MCMC techniques.

We have introduced extensions to linear and logarithmic pooling to marginals of different but overlapping quantities. Linear pooling, introduced in Section 1.2, could be extended to induce dependence between the components of *ϕ* using multivariate or vine copulas (Kurowicka and Joe, 2011; Nelsen, 2006), or other techniques (Lin et al., 2014). Copula methods are particularly appealing as, depending on the choice of copula, they yield computationally cheap to evaluate expressions for the density function, are easy to sample, and induce correlation between an arbitrary number of marginals.

Our parallel multi-stage sampler currently only considers *M* = 3 submodels, rather than the fully generic definition of chained Markov melding in Equation (10). Whilst we anticipate needing more complex methods in large *M* settings, the value of *M* at which the performance of our multi-stage sampler becomes unacceptable will depend on the specific submodels and data under consideration. A general method would consider a large and arbitrary number of submodels in a chain, and initially split the chain into more/fewer pieces depending on the computational resources available. Designing such a method is complex, as it would have to:

- avoid requiring the inverse of any component of *ϕ* with a noninvertible definition,
- estimate the relative cost of sampling each submodel’s subposterior, to split the chain of submodels into steps/jobs of approximately the same computational cost,
- decide the order in which pieces of the chain are combined.

These are substantial challenges. It may be possible to use combine the ideas in Lindsten et al. (2017) and Kuntz et al. (2021), who propose a parallel Sequential Monte Carlo method, with the aforementioned constraints to obtain a generic methodology. Ideally we would retain the ability to use existing implementations of the submodels, however the need to recompute the weights of the particles, and hence reevaluate previously considered submodels, may preclude this requirement. Our current sampler is also sensitive to large differences in location or scale of the target distribution between the stages. The impact of these differences can be ameliorated using the methodology of Manderson and Goudie (2022), and, more generally, Sequential Monte Carlo samplers are likely to perform better in these settings.

Our chained Markov melding methodology is general and permits any form of uncertainty in the common quantities. In Section 5 we use our chained melded model to incorporate uncertainty in the event times and indicators into a survival submodel. Some specific forms of uncertainty in the event times have been considered in previous work. These include Wang et al. (2020), who consider uncertain event times arising from record linkage, where the event time is assumed to be one of a finite number of event times arising from the record linkage; and Giganti et al. (2020), Oh et al. (2018), and Oh et al. (2021), who leverage external validation data to account for measurement error in the event time. However, the general and Bayesian nature of our methodology readily facilitates any form of uncertainty in the event times and the event indicators; uncertainty in the latter is not considered in the cited papers.

The example in Section 5 has three more interesting aspects to discuss. Firstly, the P/F ratio data used in the first submodel is obtained by finding all blood gas measurements from arterial blood samples. Approximately 20% of the venous/arterial labels are missing. In these instances a logistic regression model, fit by the MIMIC team^10^, is used to predict the missing label based on other covariates. It is theoretically possible to refit the model in a Bayesian framework and use the chained melded model to incorporate the uncertainty in the predicted sample label – adding another ‘link’ to the chain.

Secondly, the application of our multi-stage sampler to this example is similar to the two-stage approach used for joint longitudinal and time-to-event models (see Mauff et al., 2020, for a description of this approach). In the two-stage approach, the longitudinal model is fit using standard MCMC methods in stage one, and the samples are reused in stage two when considering the time-to-event data. This can significantly reduce the computational effort required to fit the joint model. However, unlike our multi-stage sampler, the typical two-stage approach does not target the full posterior distribution, which can lead to biased estimates (though Mauff et al. (2020) extend the typical two-stage approach to reduce this bias).

Thirdly, we observe a lack of flexibility the baseline hazard, visible in Figure 10. More complex hazards could be employed, e.g. modelling the (log-)hazard using a (penalised) B-spline (Royston and Parmar, 2002; Rosenberg, 1995; Rutherford et al., 2015). However, this increased flexibility precludes an analytic form for the survival function. Whilst numerical integration is possible it is not trivial, particularly when the hazard is discontinuous, as our hazard is at the breakpoint. Splines also have more coefficients than the single parameter of the Weibull hazard. Identifiability issues arise with a small number of individuals, many of whom are censored, and are compounded when there are a relatively large number of other parameters (*α, **θ***). Whilst we do not believe these costs are worth incurring for our example, for settings with a larger number of patients and more complicated longitudinal submodels the increased flexibility may be vital.

## Supplementary Material

Appendix

## Footnotes

1 *”Chained graphs”* were considered by Lauritzen and Richardson (2002), however they are unrelated to our proposed model. We use “chained” to emphasise the nature of the relationships between submodels.

2 This is shown in Appendix B of the online supplement to Goudie et al. (2019).

3 Some care is required if the authoritative submodel is p_*m*_ for *m ∈* {1, 2, *M* − 1, *M*}. If it is taken to be *m ∈* {1, 2}, then *g*_1_ does not exist, and additionally in the *m* = 1 case p_1_(*ϕ*_0∩1_, *ϕ*_1∩2_) ≔ p_1_(*ϕ*_1∩2_). The *m ∈* { *M* − 1, *M*} cases have analogous definitions.

4 For completeness, Appendix B describes such a sequential MCMC sampler. We do not use the sequential sampler in this paper.

5
https://doi.org/10.5281/zenodo.6552714

6 P/F data contain many outliers for, amongst many possible reasons, arterial/venous blood sample mislabelling; incorrectly recorded oxygenation support information; and differences between sample collection time, lab result time, and the observation time as recorded in the EHR.

7 We also initialise Stan at the previous value of *ψ*_2_, and disable all adaptive procedures as the default (identity) mass matrix and step size are suitable for this example.

8
https://doi.org/10.5281/zenodo.6552714

9 For each individual the samples of ${\left(T_{i},d_{i}\right)}_{i=1}^{N}$-pairs are sorted by *T_i_*, and the ${\left\lfloor \frac{N}{2}\right\rfloor }^{\text{th}}$ tuple $\left({\hat{T}}_{i},{\hat{d}}_{i}\right)$ is chosen as the median.

10 The coefficients, classification threshold, and the imputation used in the case of missing data are supplied in the blood-gasses.sql file in the GitHub repository accompanying this paper. No other information is available about this model (the data used to produce the coefficients, and the performance of the fitted model).
