# Supplementary material for: Combining chains of Bayesian models with Markov melding
Source: Bayesian Anal. Author manuscript; Available in PMC 2023 Aug 16. (PMC7614958; doi:10.1214/22-BA1327)
Supplement: Appendix [file EMS152034-supplement-Appendix.pdf]

Appendices

A Log pooling Gaussian densities

We can exactly compute p_{pool} when logarithmically pooling Gaussian densities. Noting that, in the one dimensional case, $\mathrm{N}(\phi; \mu, \sigma^2)^{\lambda_m} = \mathrm{N}(\phi; \mu, \frac{\sigma^2}{\lambda_m})$, we use the results of Bromiley (2003) and write

$$\Omega_1 = \left(\begin{bmatrix} \lambda_1^{-1} & 0 \\ 0 & \lambda_3^{-1} \end{bmatrix} \begin{bmatrix} \sigma_1^2 & 0 \\ 0 & \sigma_3^2 \end{bmatrix} \right)^{-1}, \quad \Omega_2 = \left(\begin{bmatrix} \lambda_2^{-1} & 0 \\ 0 & \lambda_2^{-1} \end{bmatrix} \begin{bmatrix} \sigma_2^2 & \rho\sigma_2^2 \\ \rho\sigma_2^2 & \sigma_2^2 \end{bmatrix} \right)^{-1} \tag{51}$$

$$\Sigma_{\text{log}} = (\Omega_1 + \Omega_2)^{-1}, \quad \mu_{\text{log}} = \Sigma_{\text{log}} \left(\Omega_1 \begin{bmatrix} \mu_1 \\ \mu_3 \end{bmatrix} + \Omega_2 \begin{bmatrix} \mu_{2,1} \\ \mu_{2,1} \end{bmatrix} \right), \tag{52}$$

hence $\mathrm{p}_{\text{pool}}(\phi_{1\cap 2}, \phi_{2\cap 3}) = \mathrm{N}((\phi_{1\cap 2}, \phi_{2\cap 3}); \mu_{\text{log}}, \Sigma_{\text{log}})$. The choice of λ_2 is critical; by controlling the contribution of p_2 to p_{pool}, λ_2 controls the degree of correlation present in the latter. The left hand column of Figure 3 illustrates this phenomena. When $\lambda_1 = \lambda_3 = 0 \implies \lambda_2 = 1$, all correlation in p_2 is present in p_{pool}. The correlation decreases for increasing values of λ_1 until $\lambda_1 = \lambda_3 = 0.5 \implies \lambda_2 = 0$, where no correlation persists.

B Sequential sampler

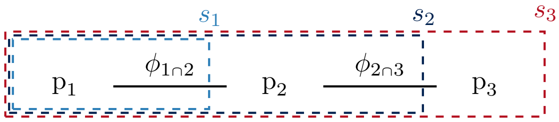

Figure 11: A DAG of the submodels and their common quantities, with the sequential sampling strategy overlaid. The stage one target (s_1) is encapsulated in the light blue dashed line; stages two and three (s_2, s_3) are in dark blue and red respectively.

Figure 11 depicts graphically the strategy employed by the sequential sampler. The sequential sampler assumes that the pooled prior decomposes such that

$$\mathrm{p}_{\text{pool}}(\boldsymbol{\phi}) = \mathrm{p}_{\text{pool},1}(\phi_{1\cap 2})\mathrm{p}_{\text{pool},2}(\phi_{1\cap 2}, \phi_{2\cap 3})\mathrm{p}_{\text{pool},3}(\phi_{1\cap 2}, \phi_{2\cap 3}). \tag{53}$$

This is necessary to avoid sampling all components of $\boldsymbol{\phi}$ in the first stage. All pooled priors trivially satisfy (53), as we can assume all but $\mathrm{p}_{\text{pool},3}(\phi_{1\cap 2}, \phi_{2\cap 3})$ are improper, flat distributions. However, including some portion of the pooled prior in each stage of the sampler can improve performance, and eliminate computational instabilities when submodel likelihoods contain little information.

Stage one

Stage one of the sequential sampler targets

$$\mathrm{p}_{\text{meld},1}(\phi_{1\cap 2}, \psi_1 \mid Y_1) \propto \mathrm{p}_{\text{pool},1}(\phi_{1\cap 2}) \frac{\mathrm{p}_1(\phi_{1\cap 2}, \psi_1, Y_1)}{\mathrm{p}_1(\phi_{1\cap 2})}, \tag{54}$$

using a generic proposal kernel for both $\phi_{1\cap 2}$ and ψ_1. The corresponding acceptance probability for a proposed update from $(\phi_{1\cap 2}, \psi_1)$ to $(\phi^*_{1\cap 2}, \psi^*_1)$ is

$$\alpha((\phi^*_{1\cap 2}, \psi^*_1), (\phi_{1\cap 2}, \psi_1)) = \frac{\mathrm{p}_{\mathrm{pool},1}(\phi^*_{1\cap 2})}{\mathrm{p}_{\mathrm{pool},1}(\phi_{1\cap 2})} \frac{\mathrm{p}_1(\phi^*_{1\cap 2}, \psi^*_1, Y_1)\mathrm{p}_1(\phi_{1\cap 2})}{\mathrm{p}_1(\phi_{1\cap 2}, \psi_1, Y_1)\mathrm{p}_1(\phi^*_{1\cap 2})} \frac{\mathrm{q}(\phi_{1\cap 2}, \psi_1 \mid \phi^*_{1\cap 2}, \psi^*_1)}{\mathrm{q}(\phi^*_{1\cap 2}, \psi^*_1 \mid \phi_{1\cap 2}, \psi_1)}. \tag{55}$$

Stage two

The stage two target augments the stage one target by including the second submodel, corresponding prior marginal distribution, and an additional pooled prior term

$$\mathrm{p}_{\mathrm{meld},2}(\phi_{1\cap 2}, \phi_{2\cap 3}, \psi_1, \psi_2 \mid Y_1, Y_2) \propto \mathrm{p}_{\mathrm{pool},1}(\phi_{1\cap 2})\mathrm{p}_{\mathrm{pool},2}(\phi_{1\cap 2}, \phi_{2\cap 3}) \frac{\mathrm{p}_1(\phi_{1\cap 2}, \psi_1, Y_1)}{\mathrm{p}_1(\phi_{1\cap 2})} \frac{\mathrm{p}_2(\phi_{1\cap 2}, \phi_{2\cap 3}, \psi_2, Y_2)}{\mathrm{p}_2(\phi_{1\cap 2}, \phi_{2\cap 3})}. \tag{56}$$

A Metropolis-within-Gibbs strategy is employed, where the stage one samples are used as a proposal for $\phi_{1\cap 2}$, whilst a generic proposal kernel is used for ψ_2 and $\phi_{2\cap 3}$. Thus the proposal distributions for $\phi^*_{1\cap 2}$ and $(\phi^*_{2\cap 3}, \psi^*_2)$ are

$$\phi^*_{1\cap 2}, \psi^*_1 \mid \phi_{2\cap 3}, \psi_2 \sim \mathrm{p}_{\mathrm{meld},1}(\phi^*_{1\cap 2}, \psi^*_1 \mid Y_1) \tag{57}$$

$$\phi^*_{2\cap 3}, \psi^*_2 \mid \phi_{1\cap 2}, \psi_1 \sim \mathrm{q}(\phi^*_{2\cap 3}, \psi^*_2 \mid \phi_{2\cap 3}, \psi_2). \tag{58}$$

The acceptance probability for this proposal strategy is

$$\alpha((\phi^*_{1\cap 2}, \psi^*_1), (\phi_{1\cap 2}, \psi_1)) = \frac{\mathrm{p}_{\mathrm{pool},2}(\phi^*_{1\cap 2}, \phi_{2\cap 3})}{\mathrm{p}_{\mathrm{pool},2}(\phi_{1\cap 2}, \phi_{2\cap 3})} \frac{\mathrm{p}_2(\phi^*_{1\cap 2}, \phi_{2\cap 3}, \psi_2, Y_2)\mathrm{p}_2(\phi_{1\cap 2}, \phi_{2\cap 3})}{\mathrm{p}_2(\phi_{1\cap 2}, \phi_{2\cap 3}, \psi_2, Y_2)\mathrm{p}_2(\phi^*_{1\cap 2}, \phi_{2\cap 3})} \tag{59}$$

$$\alpha((\phi^*_{2\cap 3}, \psi^*_2), (\phi_{2\cap 3}, \psi_2)) = \frac{\mathrm{p}_{\mathrm{pool},2}(\phi_{1\cap 2}, \phi^*_{2\cap 3})}{\mathrm{p}_{\mathrm{pool},2}(\phi_{1\cap 2}, \phi_{2\cap 3})} \frac{\mathrm{p}_2(\phi_{1\cap 2}, \phi^*_{2\cap 3}, \psi^*_2, Y_2)\mathrm{p}_2(\phi_{1\cap 2}, \phi_{2\cap 3})}{\mathrm{p}_2(\phi_{1\cap 2}, \phi_{2\cap 3}, \psi_2, Y_2)\mathrm{p}_2(\phi_{1\cap 2}, \phi^*_{2\cap 3})} \frac{\mathrm{q}(\phi_{2\cap 3}, \psi_2 \mid \phi^*_{2\cap 3}, \psi^*_2)}{\mathrm{q}(\phi^*_{2\cap 3}, \psi^*_2 \mid \phi_{2\cap 3}, \psi_2)}. \tag{60}$$

Our judicious choice of proposal distribution has resulted in a cancellation in Equation (59) which removes all terms related to p_1. Similarly, all terms related to p_1 are constant – hence cancel – in Equation (60). This eliminates any need to re-evaluate the first submodel.

Stage three

In stage three we target the full melded posterior

$$\mathrm{p}_{\mathrm{meld},3}(\phi_{1\cap 2}, \phi_{2\cap 3}, \psi_1, \psi_2, \psi_3 \mid Y_1, Y_2, Y_3) \propto \mathrm{p}_{\mathrm{meld}}(\phi_{1\cap 2}, \phi_{2\cap 3}, \psi_1, \psi_2, \psi_3 \mid Y_1, Y_2, Y_3). \tag{61}$$

The target has now been broadened to include terms from the third submodel and the entirety of the pooled prior. Again, we employ a Metropolis-within-Gibbs sampler, with proposals drawn such that

$$\phi^*_{1\cap 2}, \phi^*_{2\cap 3}, \psi^*_1, \psi^*_2 \mid \psi_3 \sim \mathrm{p}_{\mathrm{meld},2}(\phi^*_{1\cap 2}, \phi^*_{2\cap 3}, \psi^*_1, \psi^*_2 \mid Y_1, Y_2) \tag{62}$$

$$\psi^*_3 \mid \phi_{1\cap 2}, \phi_{2\cap 3}, \psi_1, \psi_2 \sim \mathrm{q}(\psi^*_3 \mid \psi_3), \tag{63}$$

which leads to acceptance probabilities of

$$\alpha((\phi^*_{1\cap 2}, \phi^*_{2\cap 3}, \psi^*_1, \psi^*_2), (\phi_{1\cap 2}, \phi_{2\cap 3}, \psi_1, \psi_2)) = \tag{64}$$
$$\frac{\mathrm{p}_{\mathrm{pool},3}(\phi^*_{1\cap 2}, \phi^*_{2\cap 3})}{\mathrm{p}_{\mathrm{pool},3}(\phi_{1\cap 2}, \phi_{2\cap 3})} \frac{\mathrm{p}_3(\phi^*_{2\cap 3}, \psi_3, Y_3)\mathrm{p}_3(\phi_{2\cap 3})}{\mathrm{p}_3(\phi_{2\cap 3}, \psi_3, Y_3)\mathrm{p}_3(\phi^*_{2\cap 3})}$$

$$\alpha\left(\psi^*_3, \psi_3\right) = \frac{\mathrm{p}_3(\phi_{2\cap 3}, \psi^*_3, Y_3)\mathrm{q}(\psi_3 \mid \psi^*_3)}{\mathrm{p}_3(\phi_{2\cap 3}, \psi_3, Y_3)\mathrm{q}(\psi^*_3 \mid \psi_3)}. \tag{65}$$

The informed choice of proposal distribution for $(\phi_{1\cap 2}, \phi_{2\cap 3}, \psi_1, \psi_2)$ has allowed us to target the full melded posterior without needing to evaluate all submodels simultaneously.

C Normal approximation calculations

Substituting in the approximations of Section 3.2 to Equation (36) yields the approximate melded posterior

$$\widehat{\mathrm{p}}_{\mathrm{meld}}(\phi_{1\cap 2}, \phi_{2\cap 3}, \psi_2 \mid Y_1, Y_2, Y_3) \propto$$
$$\frac{\widehat{\mathrm{p}}_1(\phi_{1\cap 2} \mid \widehat{\mu}_1, \widehat{\Sigma}_1)}{\widehat{\mathrm{p}}_1(\phi_{1\cap 2} \mid \widehat{\mu}_{1,0}, \widehat{\Sigma}_{1,0})} \frac{\widehat{\mathrm{p}}_3(\phi_{2\cap 3} \mid \widehat{\mu}_3, \widehat{\Sigma}_3)}{\widehat{\mathrm{p}}_3(\phi_{2\cap 3} \mid \widehat{\mu}_{3,0}, \widehat{\Sigma}_{3,0})} \mathrm{p}_2(\phi_{1\cap 2}, \phi_{2\cap 3}, \psi_2 \mid Y_2). \tag{66}$$

Noting that the product of independent normal densities is an unnormalised multivariate normal density with independent components, we rewrite Equation (66) as

$$\widehat{\mathrm{p}}_{\mathrm{meld}}(\phi_{1\cap 2}, \phi_{2\cap 3}, \psi_2 \mid Y_1, Y_2, Y_3) \propto \frac{\widehat{\mathrm{p}}_{\mathrm{nu}}(\phi_{1\cap 2}, \phi_{2\cap 3} \mid \widehat{\mu}_{\mathrm{nu}}, \widehat{\Sigma}_{\mathrm{nu}})}{\widehat{\mathrm{p}}_{\mathrm{de}}(\phi_{1\cap 2}, \phi_{2\cap 3} \mid \widehat{\mu}_{\mathrm{de}}, \widehat{\Sigma}_{\mathrm{de}})} \mathrm{p}_2(\phi_{1\cap 2}, \phi_{2\cap 3}, \psi_2 \mid Y_2),$$

$$\widehat{\mu}_{\mathrm{nu}} = \begin{bmatrix} \widehat{\mu}_1 \\ \widehat{\mu}_3 \end{bmatrix}, \quad \widehat{\Sigma}_{\mathrm{nu}} = \begin{bmatrix} \widehat{\Sigma}_1 & 0 \\ 0 & \widehat{\Sigma}_3 \end{bmatrix}, \quad \widehat{\mu}_{\mathrm{de}} = \begin{bmatrix} \widehat{\mu}_{1,0} \\ \widehat{\mu}_{3,0} \end{bmatrix}, \quad \widehat{\Sigma}_{\mathrm{de}} = \begin{bmatrix} \widehat{\Sigma}_{1,0} & 0 \\ 0 & \widehat{\Sigma}_{3,0} \end{bmatrix}. \tag{67}$$

The ratio of normal densities is also an unnormalised normal density, and hence Equation (67) simplifies to

$$\widehat{\mathrm{p}}_{\mathrm{meld}}(\phi_{1\cap 2}, \phi_{2\cap 3}, \psi_2 \mid Y_1, Y_2, Y_3) \propto \widehat{\mathrm{p}}(\phi_{1\cap 2}, \phi_{2\cap 3} \mid \widehat{\mu}, \widehat{\Sigma})\mathrm{p}_2(\phi_{1\cap 2}, \phi_{2\cap 3}, \psi_2 \mid Y_2),$$
$$\widehat{\Sigma} = \left(\widehat{\Sigma}^{-1}_{\mathrm{nu}} - \widehat{\Sigma}^{-1}_{\mathrm{de}}\right)^{-1}, \quad \widehat{\mu} = \widehat{\Sigma}\left(\widehat{\Sigma}^{-1}_{\mathrm{nu}}\widehat{\mu}_{\mathrm{nu}} - \widehat{\Sigma}^{-1}_{\mathrm{de}}\widehat{\mu}_{\mathrm{de}}\right). \tag{68}$$

D Calculating the cumulative fluid balance from the raw fluid data

In the raw fluid data each patient has $\tilde{l} = 1, \ldots, \tilde{L}_i$ observations. Each observation $\tilde{x}_{i,\tilde{l}}$ is typically a small fluid administration (e.g. an injection of some medicine in saline solution), or a fluid discharge (almost always urine excretion). The observations have corresponding observation times $\tilde{u}_{i,\tilde{l}}$, with $\tilde{\boldsymbol{u}}_i = \{\tilde{u}_{i,1}, \ldots, \tilde{u}_{i,\tilde{L}_i}\}$ and $\tilde{\boldsymbol{x}}_i = \{\tilde{x}_{i,1}, \ldots, \tilde{x}_{i,\tilde{L}_i}\}$. We code the fluid administrations/inputs as positive values, and the excretions/outputs as negative values. Each patient has an enormous number of raw fluid observations ($L_i \ll \tilde{L}_i$) and it is computationally infeasible to consider them all at once. We aggregate the raw fluid observations into 8-hourly changes in fluid balance. From these 8-hourly changes we calculate the cumulative fluid balance.

Mathematically, we define an ordered vector of boundary values

$$\boldsymbol{v}_i = (\lfloor \min\{\tilde{\boldsymbol{u}}_i\} \rfloor, \lfloor \min\{\tilde{\boldsymbol{u}}_i\} \rfloor + \frac{1}{3}, \ldots, \lceil \max\{\tilde{\boldsymbol{u}}_i\} \rceil), \tag{69}$$

noting that $\dim(\boldsymbol{v}_i) = L_i + 1$. Because the observation times encoded as *days since ICU admission* and we are interested in the 8-hourly changes, our floor and ceiling functions round down or up to the appropriate third respectively. The raw fluid observations are then divided up into L_i subsets of $\{\tilde{\boldsymbol{x}}_i, \tilde{\boldsymbol{u}}_i\}$ based on which boundary values the observation falls in between:

$$V_{i,l} = \{\{\tilde{\boldsymbol{x}}_i, \tilde{\boldsymbol{u}}_i\} \mid v_{i,l} \leq \tilde{\boldsymbol{u}}_i < v_{i,l+1}\}, \tag{70}$$

for $l = 1, \ldots, L_i$. Denote $N^V_{i,l} = |V_{i,l}| \,/\, 2$ (dividing by two as $V_{i,l}$ contains both the observation and the observation time). The lth 8-hourly fluid change $\Delta_{i,l}$ and corresponding observation time $u_{i,l}$ can then be computed as

$$\Delta_{i,l} = \sum_{s=1}^{N^V_{i,l}} \tilde{x}_{i,s}, \text{ s.t. } \tilde{x}_{i,s} \in V_{i,l}, \qquad u_{i,l} = \frac{1}{N^V_{i,l}} \sum_{s=1}^{N^V_{i,l}} \tilde{u}_{i,s}, \text{ s.t. } \tilde{u}_{i,s} \in V_{i,l}. \tag{71}$$

Finally, the 8-hourly cumulative fluid balance data are computed by $x_{i,l} = \sum_{s=1}^{l} \Delta_{i,s}$, and we assume they too are observed at $u_{i,l}$.

E Priors and justification for the cumulative fluid submodel

The parameters for the gamma prior for $\eta^b_{1,i}$ and $\eta^a_{1,i}$ are obtained by assuming that the 2.5-, 50-, and 97.5- percentiles are at 0.5, 5, and 20 (Belgorodski et al., 2017). A slope of 0.5 (i.e. the change in cumulative fluid balance per day) is unlikely but possible due to missing data; a slope of 20 is also unlikely but possible as extremely unwell patients can have very high respiratory rates and thus require large fluid inputs.

The prior for the breakpoint κ_i is derived as follows. Define $u_{i,(1)} = \min(\boldsymbol{u}_i)$ and $u_{i,(n)} = \max(\boldsymbol{u}_i)$, with $r_i = u_{i,(n)} - u_{i,(1)}$. We reparameterise the breakpoint by noting that $\kappa_i = \kappa^{\text{raw}}_i r_i + u_{i,(1)}$, where $\kappa^{\text{raw}} \in [0, 1]$. We then set $\kappa^{\text{raw}}_i \sim \text{Beta}(5, 5)$ to regularise the breakpoint towards the middle of each individual's stay in ICU. This is crucial to

ensure the submodel is identifiable when there is little evidence of a breakpoint in the data. Note that this results in the following analytic expression for $\mathrm{p}_2(\phi_{2\cap 3})$

$$\mathrm{p}_3(\phi_{2\cap 3}) = \prod_{i=1}^{N} \mathrm{p}(\eta_{1,i}^{b})\mathrm{p}(\eta_{1,i}^{a})\mathrm{p}(\kappa_i), \text{ with } \mathrm{p}(\kappa_i) = \mathrm{p}_{\kappa_i^{\mathrm{raw}}}\left(\frac{\kappa_i - u_{i,(1)}}{r_i}\right)\frac{1}{r_i} \tag{72}$$

by the change of variables formula.

Specifying a prior for $\eta_{0,i}$, the cumulative fluid balance at κ_i, is difficult because it too depends on the length of stay. Instead, we reparameterise so that $\eta_{0,i}$ is a function of the y-intercept $\eta_{0,i}^{\mathrm{raw}}$.

$$\eta_{0,i} = (\eta_{0,i}^{\mathrm{raw}} + \eta_{1,i}^{b}\kappa_i)\mathbf{1}_{\{0<\kappa_i\}} + (\eta_{0,i}^{\mathrm{raw}} + \eta_{1,i}^{a}\kappa_i)\mathbf{1}_{\{0\geq\kappa_i\}} \tag{73}$$

We place a $\mathrm{LogNormal}(1.61, 0.47^2)$ prior on $\eta_{0,i}^{\mathrm{raw}}$. These values are obtained assuming that, *a priori*, the $2.5\%, 50\%$, and 99% percentiles of $\eta_{0,i}^{\mathrm{raw}}$ are $0.5, 5$, and 15 respectively (Belgorodski et al., 2017). This is a broad prior that reflects the numerous possible admission routes into the ICU. We expect those admitted from the wards to have little pre-admission fluid data. Those admitted from the operating theatre occasionally have their in-theatre fluid input recorded after admission into the ICU, with no easy way to distinguish these records in the data.

F Analytic form for the survival function

The hazard at arbitrary time t is

$$\begin{aligned}
h_i(t) &= \gamma t^{\gamma-1}\exp\left\{\boldsymbol{w}_i^{\top}\boldsymbol{\theta} + \alpha\frac{\partial}{\partial t}m_i(t)\right\} \\
m_i(t) &= \eta_{0,i} + \eta_{1,i}^{b}(t-\kappa_i)\mathbf{1}_{\{t<\kappa_i\}} + \eta_{1,i}^{a}(t-\kappa_i)\mathbf{1}_{\{t\geq\kappa_i\}} \\
\frac{\partial}{\partial t}m_i(t) &= \eta_{1,i}^{b}\mathbf{1}_{\{t<\kappa_i\}} + \eta_{1,i}^{a}\mathbf{1}_{\{t\geq\kappa_i\}}.
\end{aligned}$$

Then, for $t > \kappa_i$, the cumulative hazard is

$$\begin{aligned}
\int_0^t h_i(u)\mathrm{d}u &= \int_0^t \gamma u^{\gamma-1}\exp\left\{\boldsymbol{w}_i^{\top}\boldsymbol{\theta} + \alpha\eta_{1,i}^{b}\mathbf{1}_{\{u<\kappa_i\}} + \alpha\eta_{1,i}^{a}\mathbf{1}_{\{u\geq\kappa_i\}}\right\}\mathrm{d}u \\
&= \gamma\exp\{\boldsymbol{w}_i^{\top}\boldsymbol{\theta}\}\int_0^t u^{\gamma-1}\exp\left\{\alpha\eta_{1,i}^{b}\mathbf{1}_{\{u<\kappa_i\}} + \alpha\eta_{1,i}^{a}\mathbf{1}_{\{u\geq\kappa_i\}}\right\}\mathrm{d}u \\
&= \gamma\exp\{\boldsymbol{w}_i^{\top}\boldsymbol{\theta}\}\left[\int_0^{\kappa_i} u^{\gamma-1}\exp\left\{\alpha\eta_{1,i}^{b}\right\}\mathrm{d}u + \int_{\kappa_i}^{t} u^{\gamma-1}\exp\left\{\alpha\eta_{1,i}^{a}\right\}\mathrm{d}u\right] \\
&= \exp\{\boldsymbol{w}_i^{\top}\boldsymbol{\theta}\}\left[\exp\left\{\alpha\eta_{1,i}^{b}\right\}\kappa_i^{\gamma} + \exp\left\{\alpha\eta_{1,i}^{a}\right\}(t^{\gamma} - \kappa_i^{\gamma})\right]
\end{aligned}$$

and for $t < \kappa_i$

$$\begin{aligned}\int_0^t h_i(u)\mathrm{d}u &= \gamma \exp\{\boldsymbol{w}_i^\top \boldsymbol{\theta}\} \left[\int_0^t u^{\gamma - 1} \exp\left\{\alpha \eta_{1,i}^{b}\right\} \mathrm{d}u \right] \\ &= \exp\{\boldsymbol{w}_i^\top \boldsymbol{\theta}\} \left[\exp\left\{\alpha \eta_{1,i}^{b}\right\} t_i^{\gamma}\right] \\ &= t_i^{\gamma} \exp\{\boldsymbol{w}_i^\top \boldsymbol{\theta} + \alpha \eta_{1,i}^{b}\}.\end{aligned}$$

The survival functions then have corresponding definitions for $t > \kappa_i$ and $t < \kappa_i$ as $S_i(t) = \exp\{-\int_0^t h_i(u)\mathrm{d}u\}$.

G Survival submodel prior justification

Our prior for $(\gamma, \alpha, \boldsymbol{\theta})$ must result in a plausible distribution for $\mathrm{p}_{2,i}(T_i \mid d_i = 1)$, and a reasonable balance between $d_i = 1$ and $d_i = 0$ events. The primary concern is unintentionally specifying a prior for which the bulk of $\mathrm{p}_{2,i}(T_i \mid d_i = 1)$ is very close to zero. In addition, certain extreme configurations of $(\gamma, \alpha, \boldsymbol{\theta})$ cause issues for the methodology of Crowther and Lambert (2013), particularly the numerical root finding and numerical integration steps. We would like to rule out such extreme configurations *a priori*. Ideally we would encode this information a joint prior for $(\gamma, \alpha, \boldsymbol{\theta})$, but specifying the appropriate correlation structure for these parameters is prohibitively challenging. Instead we focus on specifying appropriate marginals for each of γ, α, and $\boldsymbol{\theta}$, and create visual prior predictive checks (Gabry et al., 2019; Gelman et al., 2020) to ensure the induced prior for (T_i, d_i) is acceptable.

Before justifying our chosen marginal prior, we note that the $\exp\{\boldsymbol{x}_i\theta + \alpha \frac{\partial}{\partial T_i} m_i(T_i)\}$ term implies that the priors for θ and α are on the log-scale. Hence the magnitude of these parameters must be small, otherwise all event times would be very near zero or at infinity. The asymmetric effect of the transformation from the log scale also implies that symmetric priors are not obviously sensible. From these observations we deduce that θ and α must not be too large in magnitude, however if they are negative then they can be slightly larger. Hence, we specify the skew-normal priors detailed in Section 5.3, noting that the skewness parameter for α is smaller, because $\frac{\partial}{\partial T_i} m_i(T_i)$ is strictly positive and typically between 0.5 and 20, whilst $\boldsymbol{w}_i$ is standardised to be approximately standard normal. Lastly, if γ is too far away from 1 (in either direction), then the event times are very small either because the hazard increases rapidly ($\gamma \gg 1$), or because almost all of the cumulative hazard is in the neighbourhood of 0 ($\gamma \ll 1$). We specify a gamma distribution for γ with the 1th-, 50th-, and 99th-percentiles of $\mathrm{p}_2(\gamma)$ at $0.2, 1$, and 2, allowing for a wide range of hazard shapes, but removing many of the extremes.

H Estimating submodel prior marginal distributions

For p_1, we note that $\mathrm{p}_1(\phi_{1 \cap 2}) = \prod_{i=1}^{N} \mathrm{p}_{1,i}(T_i, d_i)$, and that $\mathrm{p}_{1,i}(T_i, d_i)$ conditions on each individual's length of stay (in specifying the location of the knots), as well as the range, mean, and standard deviation of the P/F data (by standardising $\tilde{z}_{i,j}$). Simple Monte Carlo samples are drawn from $\mathrm{p}_1(\phi_{1 \cap 2})$ and used to estimate $\widehat{\mathrm{p}}_1(\phi_{1 \cap 2})$. Under

the second submodel we obtain samples from $\mathrm{p}_2(\phi_{1\cap 2}, \phi_{2\cap 3})$ using the methodology of Crowther and Lambert (2013) as implemented in `simsurv` (Brilleman, 2021). These samples are use to estimate $\widehat{\mathrm{p}}_2(\phi_{1\cap 2}, \phi_{2\cap 3})$.

P/F submodel

We approximate $\mathrm{p}_1(\phi_{1\cap 2})$ using a mixture of discrete and continuous distributions, with a discrete spike at C_i for the censored events and a beta distribution for the (rescaled) event times. Monte Carlo samples of T_i and d_i are obtained from $\mathrm{p}_{1,i}(T_i, d_i)$ by drawing $\beta_{0,i}$ and $\boldsymbol{\zeta}_i$ from their respective prior distributions and then solving (46). Denoting the estimated mixture weight $\widehat{\pi}_i \in [0, 1]$, the density estimate is

$$\widehat{\mathrm{p}}_{1,i}(T_i, d_i) = \widehat{\pi}_i \,\mathrm{Beta}\left(\frac{T_i}{C_i}; \widehat{a}, \widehat{b}\right) \frac{1}{C_i} \mathbf{1}_{\{d_i=1\}} + (1 - \widehat{\pi}_i)\mathbf{1}_{\{d_i=0, T_i=C_i\}} \tag{74}$$

where $\widehat{\pi}_i, \widehat{a}_i$ and $\widehat{b}_i$ are maximum likelihood estimates obtained using the prior samples. Examples of $\widehat{\mathrm{p}}_{1,i}(T_i, d_i)$ for a subset of individuals are displayed in Figure 12.

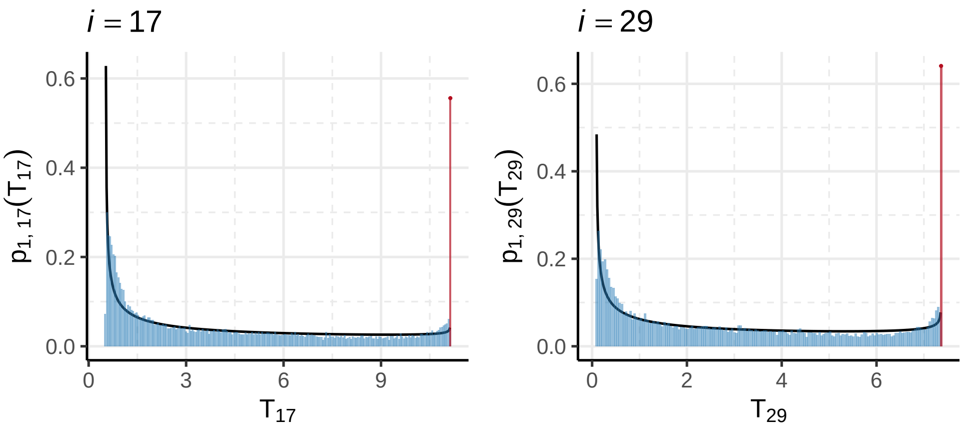

Figure 12: Fitted distribution (curve) and Monte Carlo samples drawn from $\mathrm{p}_1(\phi_{1\cap 2})$ (histogram) for a subset of the individuals in the cohort. The height of the atom at C_i (red bar and point) has been set to $1 - \widehat{\pi}_i$

Survival submodel

Our estimate of $\mathrm{p}_2(\phi_{1\cap 2}, \phi_{2\cap 3})$ relies on the fact that

$$\mathrm{p}_2(\phi_{1\cap 2}, \phi_{2\cap 3}) = \prod_{i=1}^{N} \mathrm{p}_{2,i}(T_i, d_i, \kappa_i, \eta_{1,i}^b, \eta_{1,i}^a). \tag{75}$$

As such we estimate $p_{2,i}(T_i, d_i, \kappa_i, \eta^b_{1,i}, \eta^a_{1,i})$ for each individual and take the product of these estimates. Drawing samples from $p_{2,i}(T_i, d_i, \kappa_i, \eta^b_{1,i}, \eta^a_{1,i})$ is challenging: we use the approach proposed by Crowther and Lambert (2013) as implemented in Brilleman (2021). Inspecting the samples reveals correlation between (T_i, d_i) and $(\kappa_i, \eta^b_{1,i}, \eta^a_{1,i})$ that we would like to capture in our estimate. To do so, we fit a mixture of multivariate normal distributions to transformations of the continuous parameters with support on $\mathbb{R}$,

$$\begin{gathered}\tilde{T}_i = \text{Logit}\left(\frac{T_i}{C_i}\right), \quad \tilde{\kappa}_i = \text{Logit}\left(\frac{\kappa_i - u_{i,(1)}}{u_{i,(n)} - u_{i,(1)}}\right), \\ \tilde{\eta}^b_{1,i} = \log(\eta^b_{1,i}), \quad \tilde{\eta}^a_{1,i} = \log(\eta^a_{1,i}).\end{gathered} \tag{76}$$

The resulting density estimate, with estimated mixture weight $\widehat{\theta}_i \in [0, 1]$, is

$$\begin{aligned}\widehat{p}_2(T_i, d_i, \kappa_i, \eta^b_{1,i}, \eta^a_{1,i}) = \Big[(\widehat{\theta}_i)\text{N}\left(\left[\tilde{T}_i, \tilde{\kappa}_i, \tilde{\eta}^b_{1,i}, \tilde{\eta}^a_{1,i}\right]^\top; \widehat{\mu}_{1,i}, \widehat{\Sigma}_{1,i}\right)\mathbf{1}_{\{d_i=1\}} + \\ (1 - \widehat{\theta}_i)\text{N}\left(\left[\tilde{\kappa}_i, \tilde{\eta}^b_{1,i}, \tilde{\eta}^a_{1,i}\right]^\top; \widehat{\mu}_{0,i}, \widehat{\Sigma}_{0,i}\right)\mathbf{1}_{\{d_i=0, T_i = C_i\}}\Big] J_i,\end{aligned}$$

where $\widehat{\theta}_i, \widehat{\mu}_{1,i}, \widehat{\Sigma}_{1,i}, \widehat{\mu}_{0,i}$, and $\widehat{\Sigma}_{0,i}$ are maximum likelihood estimates, and the Jacobian correction J_i is

$$J_i = \left[\left(\frac{1}{C_i - T_i} + \frac{1}{T_i}\right)^{d_i}\left(\frac{1}{u_{i,(n)} - \kappa_i} + \frac{1}{\kappa_i - u_{i,(1)}}\right)\left(\frac{1}{\eta^b_{1,i}}\right)\left(\frac{1}{\eta^a_{1,i}}\right)\right]. \tag{77}$$

We assess the fit of this estimate by drawing samples from $\widehat{p}_{2,i}(\phi_{1\cap 2}, \phi_{2\cap 3})$ and comparing them to the Monte Carlo samples drawn using `simsurv`. Our visual assessment is displayed in Figure 14 for individual $i = 19$. The normal approximation seems to fit the samples well, with the shape of $p_{2,i}(T_i \mid d_i = 1)$ closely matching that of the Monte Carlo samples, and with a similar mix of $d_i = 0$ and $d_i = 1$ samples.

We also require an estimate of $p_2(\phi_{1\cap 2})$ for experiments discussed in Section 4.7. This is obtained using the samples generated under the survival submodel prior and the methodology of Section H. The raw samples and fit are displayed in Figure 13.

I Cohort selection criteria

This appendix details the cohort selection criteria and our rationale for them. In the text we speak of the ith individual. This is because in our final data set (the data that results from applying the following criteria) we are dealing with unique individuals, however some individuals in MIMIC have multiple ICU stays. In this appendix i represents a single stay in ICU.

1. Each ICU stay must have at least 12 PaO_2/FiO_2 observations ($J_i \geq 12$), with the first 6 being greater than 350 ($z_{i,j} > 350$ for $j = 1, \ldots, 1$).

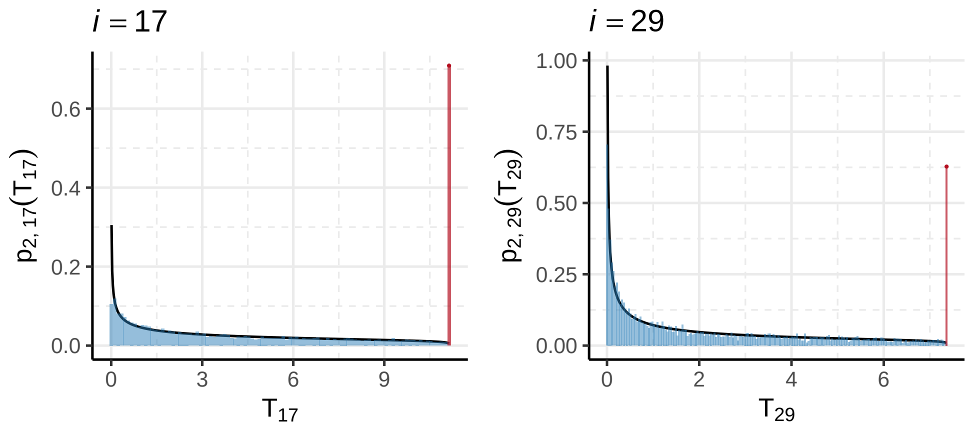

Figure 13: Fitted distribution (curve) and Monte Carlo samples drawn from $\mathrm{p}_2(\phi_{1\cap 2})$ (histogram) for a subset of the individuals in the cohort. The height of the atom at C_i (red bar and point) has been set to $1 - \widehat{\pi}_i$

- This is to ensure we have enough data to fit a B-spline with 7 internal knots. The restriction on the first 6 observations is to avoid selecting those who have already started to experience respiratory failure prior to ICU admission.

2. The time between any 2 consecutive $\mathrm{PaO_2/FiO_2}$ observations cannot exceed 2 days.

   - This is because we believe this lack of $\mathrm{PaO_2/FiO_2}$ observations is likely to be an error in the data: e.g. what appears as a single ICU stay in the data is actually two or more separate stays.

3. The fluid observations must be after ICU admission (some observations are entered as 'Pre-admission intake') and cannot be associated with fluid administered in the operating room (OR). Note that this does not mean all OR fluid administrations are removed, as some are mis-labelled.
4. There must be sufficient temporal overlap between the fluid data and the $\mathrm{PaO_2/FiO_2}$ data. Specifically,

$$\frac{\max\left\{0, \min\left[\max(\boldsymbol{t}_i), \max(\tilde{\boldsymbol{u}}_i)\right] - \max\left[\min(\boldsymbol{t}_i), \min(\tilde{\boldsymbol{u}}_i)\right]\right\}}{\max\left[\max(\boldsymbol{t}_i), \max(\tilde{\boldsymbol{u}}_i)\right] - \min\left[\min(\boldsymbol{t}_i), \min(\tilde{\boldsymbol{u}}_i)\right]} > 0.9 \qquad (78)$$

   - The numerator of (78) is strictly positive, and the denominator ensures that the quantity is bounded between 0 and 1.
   - We cannot investigate the relationship between the rate of fluid intake and respiratory failure if the latter occurs without sufficient fluid data surrounding the event.

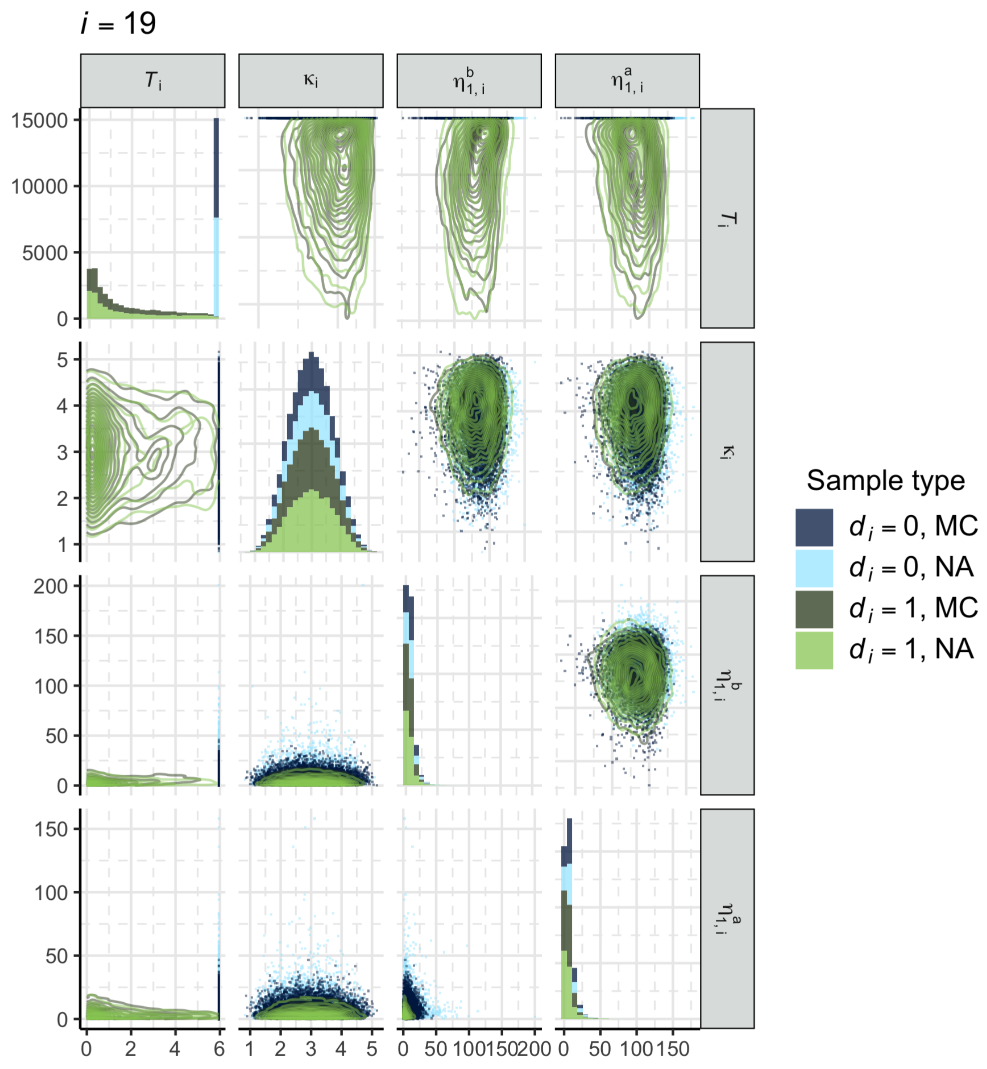

Figure 14: Monte Carlo (MC) samples from $\mathrm{p}_{2,i}(\phi_{1\cap 2}, \phi_{2\cap 3})$ obtained using `simsurv` and samples from the fitted normal approximation (NA) for $i = 19$. The panels on the off diagonal elements contain a 2D kernel density estimate for $d_i = 1$ and the samples for $d_i = 0$. Diagonal and lower-triangular panels are on their original scales, whilst the upper-triangular panels are on the log scale.

J Baseline covariate information

The baseline covariate vector $\boldsymbol{w}_i$ contains the median of the measurements taken in the first 24 hours of the ICU stay, which are then stardardised, of the following covariates:

Anion gap, Bicarbonate, Creatinine, Chloride, Glucose, Hematocrit, Hemoglobin, Platelet, Partial thromboplastin time, International normalized ratio, Prothrombin time, Sodium, blood Urea nitrogen, White blood cell count, Age at ICU admission, and Gender.

K Updating $\phi_{1\cap 2}$ and $\phi_{2\cap 3}$ in stage two individual-at-a-time

The parallel sampler described in Section 3.1 is a MH-within-Gibbs scheme, with each iteration sampling from the conditionals of the melded model

$$\begin{aligned}
&\mathrm{p}_{\mathrm{meld}}(\phi_{1\cap 2}, \psi_1 \mid \boldsymbol{Y}, \psi_2, \phi_{2\cap 3}, \psi_3), \\
&\mathrm{p}_{\mathrm{meld}}(\phi_{2\cap 3}, \psi_3 \mid \boldsymbol{Y}, \phi_{1\cap 2}, \psi_1, \psi_2), \\
&\mathrm{p}_{\mathrm{meld}}(\psi_2 \mid \boldsymbol{Y}, \phi_{1\cap 2}, \psi_1, \phi_{2\cap 3}, \psi_3).
\end{aligned} \tag{79}$$

We would like to update the first two conditionals 'individual-at-a-time'. For simplicity we will assume the pooled prior is formed using product-of-experts pooling, and focus on the first conditional $\mathrm{p}_{\mathrm{meld}}(\phi_{1\cap 2}, \psi_1 \mid \boldsymbol{Y}, \psi_2, \phi_{2\cap 3}, \psi_3)$. Similar arguments apply to the second conditional and other pooling types. Recall that

$$\begin{aligned}
\phi_{1\cap 2} &= (\phi_{1\cap 2,1}, \ldots, \phi_{1\cap 2,N}) \\
&= (\phi_{1\cap 2,1}(\chi_{1,1}), \ldots, \phi_{1\cap 2,N}(\chi_{1,N})) \\
&= ((T_1, d_1), \ldots, (T_N, d_N))\,,
\end{aligned} \tag{80}$$

and $\psi_1 = (\psi_{1,1}, \ldots, \psi_{1,N}) = (\omega_i)_{i=1}^N$. We would like to update $\phi_{1\cap 2}$ and ψ_1 by updating (T_i, d_i, ω_i) for one individual at a time, for a total of N 'sub-steps'.

Sub-step 1

Suppose we are at step $t-1$ of the Markov chain, and we are proposing values for step t. Without any loss of generality we assume that we are updating individual $i = 1$ in sub-step 1 – in practice we update the individuals in a random order for each iteration of the MH-within-Gibbs scheme.

Our target is

$$\mathrm{p}_1(\chi_{1,1}, \psi_{1,1} \mid Y_1, (\chi_{1,i}, \psi_{1,i})_{i=2}^N)\mathrm{p}_2(\phi_{1\cap 2,1}(\chi_{1,1}) \mid Y_2, (\phi_{1\cap 2,i}(\chi_{1,i}))_{i=2}^N, \psi_2, \phi_{2\cap 3}). \tag{81}$$

The model, detailed in Section 5.1, uses the conditional independence between individuals to factorise such that

$$\mathrm{p}_1(\chi_1, \psi_1 \mid Y_1) = \prod_{i=1}^N \mathrm{p}_1(\chi_{1,i}, \psi_{1,i} \mid Y_1), \tag{82}$$

which implies[11] $(\chi_{1,i}, \psi_{1,i} \perp\!\!\!\perp \chi_{1,i'}, \psi_{1,i'}) \mid Y_1$ for $i \neq i'$, hence

$$\mathrm{p}_1(\chi_{1,1}, \psi_{1,1} \mid Y_1, (\chi_{1,i}, \psi_{1,i})_{i=2}^N) = \mathrm{p}_1(\chi_{1,1}, \psi_{1,1} \mid Y_1). \tag{83}$$

[11] This property is also true of p_3.

It will be convenient to rewrite (81) as

$$\mathrm{p}_1(\chi_{1,1}, \psi_{1,1} \mid Y_1) \frac{\mathrm{p}_2(\phi_{1\cap 2,1}(\chi_{1,1}), (\phi_{1\cap 2,i}(\chi_{1,i}))_{i=2}^{N}, \psi_2, \phi_{2\cap 3} \mid Y_2)}{\mathrm{p}_2((\phi_{1\cap 2,i}(\chi_{1,i}))_{i=2}^{N}, \psi_2, \phi_{2\cap 3} \mid Y_2)}. \tag{84}$$

Suppose there are K_1 stage one samples from $\mathrm{p}_1(\chi_1, \psi_1 \mid Y_1)$, and each sample has a corresponding $\phi_{1\cap 2}$. We propose $\psi_{1,1}^*$ and $\chi_{1,1}^*$ (hence $\phi_{1\cap 2,1}^*$) by sampling a random integer k_1^* from $\{1, \ldots, K_1\}$, retrieving the corresponding values of ψ_1 and χ_1, and ignoring $(\chi_{1,2}, \ldots, \chi_{1,N}, \psi_{1,2}, \ldots, \psi_{1,N})$. Given stage one samples from the correct stationary distribution, obtained from the post-warmup samples of a well mixed set of Markov chains, such a proposal mechanism is approximately equivalent to proposing from $\mathrm{p}_1(\chi_{1,1}, \psi_{1,1} \mid Y_1)$. The quality of the approximation depends on the quality of the stage one samples. Under this proposal, noting that the denominator term in (84) does not depend on the proposed parameters, the acceptance probability for this sub-step is

$$\begin{aligned}
&\alpha\left((\chi_{1,1}^*, \psi_{1,1}^*)_{k_1^*}, (\chi_{1,1}, \psi_{1,1})\right) \\
&\quad = \frac{\mathrm{p}_1(\chi_{1,1}^*, \psi_{1,1}^* \mid Y_1)\mathrm{p}_2(\phi_{1\cap 2,1}(\chi_{1,1}^*), (\phi_{1\cap 2,i}(\chi_{1,i}))_{i=2}^{N}, \phi_{2\cap 3}, \psi_2 \mid Y_2)}{\mathrm{p}_1(\chi_{1,1}, \psi_{1,1} \mid Y_1)\mathrm{p}_2(\phi_{1\cap 2,1}(\chi_{1,1}), (\phi_{1\cap 2,i}(\chi_{1,i}))_{i=2}^{N}, \phi_{2\cap 3}, \psi_2 \mid Y_2)} \frac{\mathrm{p}_1(\chi_{1,1}, \psi_{1,1} \mid Y_1)}{\mathrm{p}_1(\chi_{1,1}^*, \psi_{1,1}^* \mid Y_1)} \\
&\quad = \frac{\mathrm{p}_2(\phi_{1\cap 2,1}(\chi_{1,1}^*), (\phi_{1\cap 2,i}(\chi_{1,i}))_{i=2}^{N}, \phi_{2\cap 3}, \psi_2 \mid Y_2)}{\mathrm{p}_2(\phi_{1\cap 2,1}(\chi_{1,1}), (\phi_{1\cap 2,i}(\chi_{1,i}))_{i=2}^{N}, \phi_{2\cap 3}, \psi_2 \mid Y_2)},
\end{aligned} \tag{85}$$

which does not depend on p_1. We store the value of k_1^* associated with the proposal (if it is accepted) in order to resample the stage one values of $\psi_{1,1}$ to reflect the information in the other submodels.

Sub-step n

At sub-step n, for $1 < n \leq N$ with $(\chi_{1,i})_{i=N+1}^{N} = \varnothing$, we have updated $((\chi_{1,i}, \psi_{1,i})_{i=1}^{n-1})$ to $((\chi_{1,i}^{[t]}, \psi_{1,i}^{[t]})_{i=1}^{n-1})$. Thus the target is

$$\begin{aligned}
&\mathrm{p}_1\left(\chi_{1,n}, \psi_{1,n} \mid Y_1, (\chi_{1,i}^{[t]}, \psi_{1,i}^{[t]})_{i=1}^{n-1}, (\chi_{1,i}, \psi_{1,i})_{i=n+1}^{N}\right) && (86)\\
&\quad \times \mathrm{p}_2\left(\phi_{1\cap 2,n}(\chi_{1,n}) \mid Y_2, \psi_2, \phi_{2\cap 3}, (\phi_{1\cap 2,i}(\chi_{1,i}^{[t]}))_{i=1}^{n-1}, (\phi_{1\cap 2,i}(\chi_{1,i}))_{i=n+1}^{N}\right) \\
&= \mathrm{p}_1\left(\chi_{1,n}, \psi_{1,n}, \mid Y_1\right) \frac{\mathrm{p}_2\left(\phi_{1\cap 2,n}(\chi_{1,n}), \psi_2, \phi_{2\cap 3}, (\phi_{1\cap 2,i}(\chi_{1,i}^{[t]}))_{i=1}^{n-1}, (\phi_{1\cap 2,i}(\chi_{1,i}))_{i=n+1}^{N} \mid Y_2\right)}{\mathrm{p}_2\left(\psi_2, \phi_{2\cap 3}, (\phi_{1\cap 2,i}(\chi_{1,i}^{[t]}))_{i=1}^{n-1}, (\phi_{1\cap 2,i}(\chi_{1,i}))_{i=n+1}^{N} \mid Y_2\right)} && (87)
\end{aligned}$$

We propose $\chi^*_{1,n}, \psi^*_{1,n}$ in the same manner as the previous section. The corresponding acceptance probability is

$$\begin{aligned}
\alpha((\chi^*_{1,n}, \psi^*_{1,n}),&(\chi_{1,n}, \psi_{1,n})) \\
&= \frac{\mathrm{p}_1\left(\chi^*_{1,n}, \psi^*_{1,n} \mid Y_1\right)}{\mathrm{p}_1\left(\chi_{1,n}, \psi_{1,n} \mid Y_1\right)} \\
&\quad \times \frac{\mathrm{p}_2(\phi_{1\cap 2,n}(\chi^*_{1,n}), \psi_2, \phi_{2\cap 3}, (\chi^{[t]}_{1,i})_{i=1}^{n-1}, (\chi_{1,i})_{i=n+1}^{N} \mid Y_2)}{\mathrm{p}_2(\phi_{1\cap 2,n}(\chi_{1,n}), \psi_2, \phi_{2\cap 3}, (\chi^{[t]}_{1,i})_{i=1}^{n-1}, (\chi_{1,i})_{i=n+1}^{N} \mid Y_2)} \\
&\quad \times \frac{\mathrm{p}_1(\chi_{1,n}, \psi_{1,n} \mid Y_1)}{\mathrm{p}_1(\chi^*_{1,n}, \psi^*_{1,n} \mid Y_1)} \\
&= \frac{\mathrm{p}_2(\phi_{1\cap 2,n}(\chi^*_{1,n}), \psi_2, \phi_{2\cap 3}, (\chi^{[t]}_{1,i})_{i=1}^{n-1}, (\chi_{1,i})_{i=n+1}^{N} \mid Y_2)}{\mathrm{p}_2(\phi_{1\cap 2,n}(\chi_{1,n}), \psi_2, \phi_{2\cap 3}, (\chi^{[t]}_{1,i})_{i=1}^{n-1}, (\chi_{1,i})_{i=n+1}^{N} \mid Y_2)}.
\end{aligned} \tag{88}$$

References

Abadi, F., Gimenez, O., Ullrich, B., Arlettaz, R., and Schaub, M. (2010). "Estimation of Immigration Rate Using Integrated Population Models." *Journal of Applied Ecology*, 47(2): 393–400. 3, 15, 17

Abbas, A. E. (2009). "A Kullback-Leibler View of Linear and Log-Linear Pools." *Decision Analysis*. 11

Ades, A. E. and Sutton, A. J. (2006). "Multiparameter Evidence Synthesis in Epidemiology and Medical Decision-Making: Current Approaches." *Journal of the Royal Statistical Society: Series A (Statistics in Society)*, 169(1): 5–35. 2

Belgorodski, N., Greiner, M., Tolksdorf, K., and Schueller, K. (2017). "rriskDistributions: Fitting Distributions to given Data or Known Quantiles." R package version 2.1.2. 32, 33

Besbeas, P., Freeman, S. N., Morgan, B. J. T., and Catchpole, E. A. (2002). "Integrating Mark–Recapture–Recovery and Census Data to Estimate Animal Abundance and Demographic Parameters." *Biometrics*, 58(3): 540–547. 2

Brilleman, S. (2021). "Simsurv: Simulate Survival Data." R package version 1.0.0. 35, 36

Brilleman, S. L., Elci, E. M., Novik, J. B., and Wolfe, R. (2020). "Bayesian Survival Analysis Using the Rstanarm R Package." *arXiv:2002.09633 [stat]*. 22

Bromiley, P. (2003). "Products and Convolutions of Gaussian Probability Density Functions." *Tina-Vision Memo*, 3(4): 1. 15, 29

Brooks, S. P., King, R., and Morgan, B. J. T. (2004). "A Bayesian Approach to Combining Animal Abundance and Demographic Data." *Animal Biodiversity and Conservation*, 27(1). 2

Burke, D. L., Ensor, J., and Riley, R. D. (2017). "Meta-Analysis Using Individual Participant Data: One-Stage and Two-Stage Approaches, and Why They May Differ." *Statistics in Medicine*, 36(5): 855–875. 14

Carpenter, B., Gelman, A., Hoffman, M., Lee, D., Goodrich, B., Betancourt, M., Brubaker, M., Guo, J., Li, P., and Riddell, A. (2017). "Stan: A Probabilistic Programming Language." *Journal of Statistical Software*, 76(1): 1–32. 18

Carvalho, L. M., Villela, D. A. M., Coelho, F. C., and Bastos, L. S. (2022). "Bayesian Inference for the Weights in Logarithmic Pooling." *Bayesian Analysis*, 1–29. 11

Crowther, M. J. and Lambert, P. C. (2013). "Simulating Biologically Plausible Complex Survival Data." *Statistics in Medicine*, 32(23): 4118–4134. 34, 35, 36

Dawid, A. P. and Lauritzen, S. L. (1993). "Hyper Markov Laws in the Statistical Analysis of Decomposable Graphical Models." *The Annals of Statistics*, 21(3): 1272–1317. 5

de Valpine, P., Turek, D., Paciorek, C. J., Anderson-Bergman, C., Lang, D. T., and Bodik, R. (2017). "Programming with Models: Writing Statistical Algorithms for General Model Structures with NIMBLE." *Journal of Computational and Graphical Statistics*, 26(2): 403–413. 18

Donnat, C., Miolane, N., Bunbury, F., and Kreindler, J. (2020). "A Bayesian Hierarchical Network for Combining Heterogeneous Data Sources in Medical Diagnoses." In *Proceedings of the Machine Learning for Health NeurIPS Workshop*, volume 136 of *Proceedings of Machine Learning Research*, 53–84. PMLR. 2

Finke, A., King, R., Beskos, A., and Dellaportas, P. (2019). "Efficient Sequential Monte Carlo Algorithms for Integrated Population Models." *Journal of Agricultural, Biological and Environmental Statistics*, 24(2): 204–224. 15, 17

Gabry, J., Mahr, T., Bürkner, P.-C., Modrák, M., Barrett, M., Weber, F., Sroka, E. C., and Vehtari, A. (2021). "Bayesplot: Plotting for Bayesian Models." R package version 1.8.0. 18

Gabry, J., Simpson, D., Vehtari, A., Betancourt, M., and Gelman, A. (2019). "Visualization in Bayesian Workflow." *Journal of the Royal Statistical Society: Series A (Statistics in Society)*, 182(2): 389–402. 11, 34

Gelman, A., Vehtari, A., Simpson, D., Margossian, C. C., Carpenter, B., Yao, Y., Kennedy, L., Gabry, J., Bürkner, P.-C., and Modrák, M. (2020). "Bayesian Workflow." *arXiv:2011.01808 [stat]*. 11, 34

Genest, C., McConway, K. J., and Schervish, M. J. (1986). "Characterization of Externally Bayesian Pooling Operators." *The Annals of Statistics*, 14(2): 487–501. 6

Giganti, M. J., Shaw, P. A., Chen, G., Bebawy, S. S., Turner, M. M., Sterling, T. R., and Shepherd, B. E. (2020). "Accounting for Dependent Errors in Predictors and Time-to-Event Outcomes Using Electronic Health Records, Validation Samples, and Multiple Imputation." *Annals of Applied Statistics*, 14(2): 1045–1061. 27

Goudie, R. J. B., Presanis, A. M., Lunn, D., De Angelis, D., and Wernisch, L. (2019). "Joining and Splitting Models with Markov Melding." *Bayesian Analysis*, 14(1): 81–109. 2, 5, 7, 23

Hastie, T. and Tibshirani, R. (1999). *Generalized Additive Models*. Boca Raton, Fla: Chapman & Hall/CRC. 20

Hinton, G. E. (2002). "Training Products of Experts by Minimizing Contrastive Divergence." *Neural Computation*, 14(8): 1771–1800. 6, 9

Hooten, M. B., Johnson, D. S., and Brost, B. M. (2019). "Making Recursive Bayesian Inference Accessible." *The American Statistician*, 1–10. 11

Jackson, D. and White, I. R. (2018). "When Should Meta-Analysis Avoid Making Hidden Normality Assumptions?" *Biometrical Journal*, 60(6): 1040–1058. 3

Johnson, A. E. W., Pollard, T. J., Shen, L., Lehman, L.-w. H., Feng, M., Ghassemi, M., Moody, B., Szolovits, P., Anthony Celi, L., and Mark, R. G. (2016). "MIMIC-III, a Freely Accessible Critical Care Database." *Scientific Data*, 3(1): 160035. 19

Kay, M. (2020). "Tidybayes: Tidy Data and Geoms for Bayesian Models." R package version 2.0.2. 18

Kedem, B., De Oliveira, V., and Sverchkov, M. (2017). *Statistical Data Fusion*. World Scientific. 2

Kharratzadeh, M. (2017). "Splines in Stan." *Stan Case Studies*, 4. 20

Kuntz, J., Crucinio, F. R., and Johansen, A. M. (2021). "The Divide-and-Conquer Sequential Monte Carlo Algorithm: Theoretical Properties and Limit Theorems." *arXiv:2110.15782 [math, stat]*. 27

Kurowicka, D. and Joe, H. (eds.) (2011). *Dependence Modeling: Vine Copula Handbook*. Singapore: World Scientific. 27

Lahat, D., Adali, T., and Jutten, C. (2015). "Multimodal Data Fusion: An Overview of Methods, Challenges, and Prospects." *Proceedings of the IEEE*, 103(9): 1449–1477. 2

Lauritzen, S. L. and Richardson, T. S. (2002). "Chain Graph Models and Their Causal Interpretations." *Journal of the Royal Statistical Society: Series B (Statistical Methodology)*, 64(3): 321–348. 2

Lebreton, J.-D., Burnham, K. P., Clobert, J., and Anderson, D. R. (1992). "Modeling Survival and Testing Biological Hypotheses Using Marked Animals: A Unified Approach with Case Studies." *Ecological Monographs*, 62(1): 67–118. 2

Lin, G., Dou, X., Kuriki, S., and Huang, J.-S. (2014). "Recent Developments on the Construction of Bivariate Distributions with Fixed Marginals." *Journal of Statistical Distributions and Applications*, 1(1): 14. 27

Lindsten, F., Johansen, A. M., Naesseth, C. A., Kirkpatrick, B., Schön, T. B., Aston, J. A. D., and Bouchard-Côté, A. (2017). "Divide-and-Conquer with Sequential Monte Carlo." *Journal of Computational and Graphical Statistics*, 26(2): 445–458. 27

Lu, C. J. and Meeker, W. Q. (1993). "Using Degradation Measures to Estimate a Time-to-Failure Distribution." *Technometrics*, 35(2): 161–174. 4

Lunn, D., Barrett, J., Sweeting, M., and Thompson, S. (2013). "Fully Bayesian Hierarchical Modelling in Two Stages, with Application to Meta-Analysis." *Journal of the Royal Statistical Society Series C*, 62(4): 551–572. 3, 11

Lunn, D., Spiegelhalter, D., Thomas, A., and Best, N. (2009). "The BUGS Project: Evolution, Critique and Future Directions." *Statistics in Medicine*, 28(25): 3049–3067. 18

Manderson, A. A. and Goudie, R. J. B. (2022). "A Numerically Stable Algorithm for Integrating Bayesian Models Using Markov Melding." *Statistics and Computing*, 32(2): 24. 27

Massa, M. S. and Lauritzen, S. L. (2010). "Combining Statistical Models." In *Algebraic Methods in Statistics and Probability II*, volume 516 of *Contemp. Math.*, 239–259. Amer. Math. Soc., Providence, RI. 5

Mauff, K., Steyerberg, E., Kardys, I., Boersma, E., and Rizopoulos, D. (2020). "Joint Models with Multiple Longitudinal Outcomes and a Time-to-Event Outcome: A Corrected Two-Stage Approach." *Statistics and Computing*, 30(4): 999–1014. 3, 4, 11, 28

Maunder, M. N. and Punt, A. E. (2013). "A Review of Integrated Analysis in Fisheries Stock Assessment." *Fisheries Research*, 142: 61–74. 2

Meng, X.-L. (2014). "A Trio of Inference Problems That Could Win You a Nobel Prize in Statistics (If You Help Fund It)." In Lin, X., Genest, C., Banks, D. L., Molenberghs, G., Scott, D. W., and Wang, J.-L. (eds.), *Past, Present, and Future of Statistical Science*, 561–586. Chapman and Hall/CRC. 3

Nelsen, R. B. (2006). *An Introduction to Copulas*. Springer New York, second edition. 27

Nicholson, G., Blangiardo, M., Briers, M., Diggle, P. J., Fjelde, T. E., Ge, H., Goudie, R. J. B., Jersakova, R., King, R. E., Lehmann, B. C. L., Mallon, A.-M., Padellini, T., Teh, Y. W., Holmes, C., and Richardson, S. (2021). "Interoperability of Statistical Models in Pandemic Preparedness: Principles and Reality." *Statistical Science (forthcoming)*. 3

NIMBLE Development Team (2019). "NIMBLE: MCMC, Particle Filtering, and Programmable Hierarchical Modeling." R package manual version 0.9.0. 18

Oh, E. J., Shepherd, B. E., Lumley, T., and Shaw, P. A. (2018). "Considerations for Analysis of Time-to-Event Outcomes Measured with Error: Bias and Correction with SIMEX." *Statistics in medicine*, 37(8): 1276–1289. 27

— (2021). "Raking and Regression Calibration: Methods to Address Bias from Correlated Covariate and Time-to-Event Error." *Statistics in Medicine*, 40(3): 631–649. 27

O'Hagan, A., Buck, C., Daneshkhah, A., Eiser, J., Garthwaite, P., Jenkinson, D., Oakley, J., and Rakow, T. (2006). *Uncertain Judgements: Eliciting Experts' Probabilities*. Statistics in Practice. Wiley. 6

Parsons, J., Niu, X., and Bao, L. (2021). "A Bayesian Hierarchical Modeling Approach to Combining Multiple Data Sources: A Case Study in Size Estimation." *arXiv:2012.05346 [stat]*. 2

Plummer, M. (2019). "Rjags: Bayesian Graphical Models Using MCMC." R package version 4-10. 18

Presanis, A. M., Pebody, R. G., Birrell, P. J., Tom, B. D. M., Green, H. K., Durnall, H., Fleming, D., and De Angelis, D. (2014). "Synthesising Evidence to Estimate Pandemic (2009) A/H1N1 Influenza Severity in 2009–2011." *Annals of Applied Statistics*, 8(4): 2378–2403. 2

Rizopoulos, D. (2012). *Joint Models for Longitudinal and Time-to-Event Data: With Applications in R*. CRC Press. 4

Rosenberg, P. S. (1995). "Hazard Function Estimation Using B-splines." *Biometrics*, 51(3): 874–887. 28

Royston, P. and Parmar, M. K. B. (2002). "Flexible Parametric Proportional-Hazards and Proportional-Odds Models for Censored Survival Data, with Application to Prognostic Modelling and Estimation of Treatment Effects." *Statistics in Medicine*, 21(15): 2175–2197. 28

Rufo, M. J., Martín, J., and Pérez, C. J. (2012a). "Log-Linear Pool to Combine Prior Distributions: A Suggestion for a Calibration-Based Approach." *Bayesian Analysis*, 7(2): 411–438. 11

Rufo, M. J., Pérez, C. J., and Martín, J. (2012b). "A Bayesian Approach to Aggregate Experts' Initial Information." *Electronic Journal of Statistics*, 6(none): 2362–2382. 11

Rutherford, M. J., Crowther, M. J., and Lambert, P. C. (2015). "The Use of Restricted Cubic Splines to Approximate Complex Hazard Functions in the Analysis of Time-to-Event Data: A Simulation Study." *Journal of Statistical Computation and Simulation*, 85(4): 777–793. 28

Schaub, M. and Abadi, F. (2011). "Integrated Population Models: A Novel Analysis Framework for Deeper Insights into Population Dynamics." *Journal of Ornithology*, 152(1): 227–237. 2

Schaub, M., Ullrich, B., Knötzsch, G., Albrecht, P., and Meisser, C. (2006). "Local Population Dynamics and the Impact of Scale and Isolation: A Study on Different Little Owl Populations." *Oikos*, 115(3): 389–400. 3, 15

Seethala, R. R., Hou, P. C., Aisiku, I. P., Frendl, G., Park, P. K., Mikkelsen, M. E., Chang, S. Y., Gajic, O., and Sevransky, J. (2017). "Early Risk Factors and the Role of Fluid Administration in Developing Acute Respiratory Distress Syndrome in Septic Patients." *Annals of Intensive Care*, 7(1): 11. 21

Soetaert, K., Hindmarsh, A. C., Eisenstat, S. C., Moler, C., Dongarra, J., and Saad, Y. (2020). "Rootsolve: Nonlinear Root Finding, Equilibrium and Steady-State Analysis of Ordinary Differential Equations." R package version 1.8.2.1. 20

Stan Development Team (2021). "RStan: The R Interface to Stan." R package version 2.26. 18

The ARDS Definition Task Force (2012). "Acute Respiratory Distress Syndrome: The Berlin Definition." *JAMA*, 307(23): 2526–2533. 4

Tom, J. A., Sinsheimer, J. S., and Suchard, M. A. (2010). "Reuse, Recycle, Reweigh: Combating Influenza through Efficient Sequential Bayesian Computation for Massive Data." *The Annals of Applied Statistics*, 4(4): 1722–1748. 11

Vehtari, A., Gelman, A., Simpson, D., Carpenter, B., and Bürkner, P.-C. (2020). "Rank-Normalization, Folding, and Localization: An Improved $\widehat{R}$ for Assessing Convergence of MCMC." *Bayesian Analysis*. 18, 23

Wang, W., Aseltine, R., Chen, K., and Yan, J. (2020). "Integrative Survival Analysis with Uncertain Event Times in Application to a Suicide Risk Study." *Annals of Applied Statistics*, 14(1): 51–73. 27

Wang, W. and Yan, J. (2021). "Shape-Restricted Regression Splines with R Package Splines2." *Journal of Data Science*, 19(3): 498–517. 20

Zipkin, E. F. and Saunders, S. P. (2018). "Synthesizing Multiple Data Types for Biological Conservation Using Integrated Population Models." *Biological Conservation*, 217: 240–250. 2, 3
